# Distinct clinical and somatic mutational features of breast tumors with high-, low-, or non-expressing human epidermal growth factor receptor 2 status

**DOI:** 10.1186/s12916-022-02346-9

**Published:** 2022-04-29

**Authors:** Guochun Zhang, Chongyang Ren, Cheukfai Li, Yulei Wang, Bo Chen, Lingzhu Wen, Minghan Jia, Kai Li, Hsiaopei Mok, Li Cao, Xiaoqing Chen, Jiali Lin, Guangnan Wei, Yingzhi Li, Yuchen Zhang, Charles M. Balch, Ning Liao

**Affiliations:** 1 Department of Breast Surgery, Guangdong Provincial People’s Hospital and Guangdong Academy of Medical Sciences, 106 Zhongshan Er Road, Guangzhou, 510080 China; 2 Foshan Women and Children Hospital, Foshan, China; 3 Department of Breast Surgery, Nanhai Second People’s Hospital, Foshan, China; 4 School of Medicine, South China University of Technology, Guangzhou, China; 5 Shantou University Medical College, Shantou, China; 6 Department of Surgical Oncology, UT MD Anderson Cancer Center, Houston, TX USA

**Keywords:** Breast cancer, Human epidermal growth factor receptor 2 (HER2), Genomic alteration, HER2-low, Next-generation sequencing (NGS), Targeted therapy

## Abstract

**Background:**

HER2-low breast cancers were reported to have distinct clinicopathological characteristics from HER2-zero; however, the difference in their genetic features remains unclear. This study investigated the clinical and molecular features of breast tumors according to HER2 status.

**Methods:**

We analyzed the clinicopathological and genomic data of 523 Chinese women with breast cancer. Genomic data was generated by targeted next-generation sequencing (NGS) of breast tumor samples using a commercial 520 gene panel. The cohort was stratified according to HER2 status as HER2-zero (*n* = 90), HER2-low (*n* = 231), and HER2-positive (*n* = 202) according to their immunohistochemistry and fluorescence in situ hybridization results.

**Results:**

HER2-low breast tumors were enriched with hormone receptor-positive tumors, and who had lower Ki67 expression levels. Genes were differentially mutated across HER2 subgroups. HER2-low tumors had significantly more mutations involved in PI3K-Akt signaling than HER2-positive (*p* < 0.001) and HER2-zero breast tumors (*p* < 0.01). HER2-zero tumors had more mutations in checkpoint factors (*p* < 0.01), Fanconi anemia (*p* < 0.05), and p53 signaling and cell cycle pathway (*p* < 0.05) compared to HER2-low breast tumors. Compared with HER2-zero tumors, HER2-low tumors had significantly lower pathological complete response rates after neoadjuvant therapy (15.9% vs. 37.5%, *p* = 0.042) and proportion of relapsed/progressed patients across follow-up time points (*p* = 0.031), but had comparable disease-free survival (*p* = 0.271).

**Conclusion:**

Our results demonstrate the distinct clinical and molecular features and clinical outcomes of HER2-low breast tumors.

**Supplementary Information:**

The online version contains supplementary material available at 10.1186/s12916-022-02346-9.

## Background

The overexpression of the human epidermal growth factor receptor 2 (HER2) is associated with shorter overall survival and higher risk of disease recurrence. HER2 overexpression is detected in 15–40% of unselected patients with breast cancer [1–4]. Genetic alterations in HER2, also known as Erb-B2 receptor tyrosine kinase 2 (ERBB2), which bring about protein overexpression, have become important biomarkers of response to HER2-targeting agents, including trastuzumab, ado-trastuzumab emtansine (T-DM1), and lapatinib [5–11]. These HER2-targeted therapies have substantially improved the clinical outcomes and survival rates of patients with HER2-positive breast cancer [5–11].

Currently, HER2 status is assayed using standard molecular methods based on the assessment of HER2 protein overexpression by immunohistochemistry (IHC) and estimation of *HER2* gene amplification by fluorescence in situ hybridization (FISH). The results from these molecular methods were assessed based on the algorithm recommended by an expert panel from the American Society of Clinical Oncology (ASCO) and the College of American Pathologists (CAP) [12–14]. The most recent update (2018) of this algorithm refined the IHC and FISH criteria into a binary classification system as either HER2-positive or HER2-negative, which eliminated the definition of HER2 equivocal status. This in turn, led to a considerable increase in HER2 FISH-negative cases and a slight decrease in HER2 FISH-positive cases [15, 16].

However, the clinical implications of classifying HER2 equivocal breast tumors as HER2-negative remain unclear. Some studies have demonstrated similar clinical and survival outcomes between HER2 equivocal and HER2-negative/zero breast tumors [15, 16], whereas other studies have revealed distinct clinical, molecular, and survival outcomes between HER2-low and HER2-zero breast tumors [17–20]. A recent report that analyzed a cohort of 2310 patients pooled from four clinical trials demonstrated the distinct biology, clinicopathological features, therapeutic response, and clinical outcome of HER2-low breast cancers (defined by IHC 1+/2+ with FISH-negative result) as compared with HER2-zero, suggesting that HER2-low should be recognized as a new subtype of breast cancer [21].

At present, novel HER2-targeting therapies with mechanisms independent of overexpressed HER2 protein have been developed. These include neratinib, a pan-EGFR tyrosine kinase inhibitor (TKI) [22], and trastuzumab deruxtecan (T-DXd, formerly DS8201a), a novel antibody-drug conjugate (ADC), which elicited 40% objective response for patients with HER2-low metastatic breast cancer [23–25]. Other novel treatments targeting HER2 protein include HER2 vaccines and bispecific antibodies [26–28]. These therapeutic advances raise an important question about the need to reclassify HER2-low as a separate entity. They also highlight the need for alternative methods to comprehensively evaluate HER2 expression and its alteration status to identify patients who might benefit from these novel targeted therapies. Therefore, it is essential to elucidate the underlying genetic background of breast tumors with HER2-zero, HER2-low, or HER2-positive expression status to gain a better understanding of their distinct features. Contemporary diagnostic methods, such as next-generation sequencing (NGS), can be explored to simultaneously evaluate *HER2* gene amplification status and other concomitant gene alterations.

We conducted this study to investigate the genomic characteristics of breast tumors with HER2-zero (IHC 0), HER2-low (IHC 1+ or IHC 2+/FISH-negative), and HER2-positive (IHC 3+ or IHC 2+/FISH-positive) status, by retrospectively analyzing the molecular, clinical, and survival data from 523 Chinese women with breast cancer. All breast tumor tissues were subjected to targeted NGS using a large gene panel to comprehensively elucidate their somatic mutational landscape. We then compared the breast cancer subtypes, molecular features, treatment outcome, and disease-free survival data according to HER2 expression status.

## Methods

### Patient selection

Women diagnosed with breast cancer at Guangdong Provincial People’s Hospital (GDPH) were prospectively recruited for genomic studies and voluntarily submitted samples for NGS as previously described [29]. The study cohort was unselected for clinical or pathological features; however, clinical and molecular data were only retrieved for the patients who met the following study inclusion criteria: (1) patients who were treatment naïve and have not received any systemic therapy; (2) patients with NGS results generated using a 520-gene targeted panel from DNA extracted from formalin-fixed paraffin-embedded (FFPE) tissue sample of the primary breast cancer obtained either by excisional biopsy or core-needle biopsy; (3) available data for HER2 status determined by IHC and/or FISH; (4) complete clinical and pathological results, including estrogen receptor (ER), progesterone receptor (PR), and Ki-67 status. IHC-based surrogate molecular subtypes were classified according to the St. Gallen 2013 guideline [30]. Patients who did not meet all the inclusion criteria were excluded. All the patients provided written informed consent for NGS-based genomic testing. This study was conducted according to the ethical principles of the Declaration of Helsinki, with the study protocol reviewed and approved by the GDPH Ethics Committee (approval no. GDREC2014122H).

### Standard HER2 testing

Thin FFPE sections of breast tumor samples from all patients were subjected to initial HER2 testing with IHC using HercepTest (Dako/Agilent, Santa Clara, CA, USA). HER2-positivity was determined as an IHC result of 3+, defined as having >10% intact membrane staining. With an IHC result of 2+ or upon patient request, the slides containing a thin section from the same breast tumor sample of the patient were submitted for subsequent FISH-based HER2 testing using HER2 IQFISH pharmDx (Dako/Agilent, Santa Clara, CA, USA). FISH-based *HER2* amplification positivity was defined as HER2 to chromosome 17 centromere (CEP17) signal ratio of ≥2.0 with HER2 signals exceeding 6.0 per cell. The slides were scored by experienced pathologists and/or technicians according to the 2018 ASCO/CAP guidelines [14].

### Tissue DNA extraction and capture-based targeted DNA sequencing

Genomic DNA was extracted from FFPE breast tumor tissue with at least 10% tumor content using QIAamp DNA FFPE tissue kit as per the manufacturer’s instructions (Qiagen, Valencia, CA, USA). DNA concentration was determined using Qubit dsDNA high-sensitivity quantification assay on the Qubit fluorometer according to the manufacturer’s instructions (Life Technologies, Carlsbad, CA, USA). Target capture of the tissue DNA samples was performed using a 520-gene panel (OncoScreen Plus, Burning Rock Biotech, Guangzhou, China) following previously published protocols [29, 31, 32]. The 520-gene panel covered 1.64 megabases (Mb) of the human genome and consisted of whole exons of 312 genes and critical exons, introns, and promoter regions of the remaining 208 genes. Briefly, tissue DNA was acoustically fragmented, with DNA fragments of 200–400 bp purified, amplified, and hybridized with capture probes to construct the NGS library. After assessing the quality and fragment size, the indexed samples were sequenced on a Nextseq500 sequencer (Illumina, Inc., San Diego, CA, USA) with paired-end reads at a target average sequencing depth of 1000×. Bioinformatics pipeline developed by Burning Rock Biotech was used to analyze the sequencing data. First, the sequencing reads were aligned to the human reference genome (version hg19) using Burrows-Wheeler aligner (version 0.7.10). Local alignment optimization, variant calling, and annotation of single-nucleotide variations (SNVs) and small insertion-deletions (Indels) were performed using GATK (version 3.2; Broad Institute, Cambridge, MA, USA) and VarScan (version 2.4.3; Genome Institute, Washington University, USA). Analysis of structural variations (SVs) was performed using Factera version 1.4.3. Copy number variations (CNVs) were analyzed based on the depth of coverage. Sequencing bias arising from guanine-cytosine (G-C) content and probe design was corrected against the coverage data. The sequencing coverage of different samples was normalized to comparable scales using the average coverage of all captured regions. CNV was called when the coverage data of the gene region was statistically different from its reference control and with a *p*-value of <0.005 and a ratio of >0.5. The normal copy number is set at 2 as per the normal diploid genome, copy number ≤1.5 is considered copy number deletion, and copy number ≥2.75 is considered copy number amplification.

### Tumor mutation burden (TMB) calculation

TMB per patient was computed as a ratio between the total number of non-synonymous mutations detected and the total coding region size of the panel using the equation below. The mutation count included non-synonymous SNVs and Indels detected within the coding region and ±2 bp upstream or downstream region and does not include hot mutation events, CNVs, SVs, and germline single-nucleotide polymorphisms (SNPs). Only mutations with allelic fractions (AF) of ≥ 2% for tissue samples and ≥ 0.2% for plasma samples were included in the mutation count. For accurate TMB calculation, maximum AF (maxAF) was calculated as ≥ 5% for tissue samples and ≥ 1% for plasma samples. The total size of the coding region for estimating TMB was 1.003 Mb for the 520-gene OncoScreen Plus panel.

$$\mathrm{TMB}=\frac{\mathrm{mutation}\ \mathrm{count}\ \left(\mathrm{except}\ \mathrm{for}\ \mathrm{CNV},\mathrm{SV},\mathrm{SNPs},\mathrm{and}\ \mathrm{hot}\ \mathrm{mutations}\right)}{1.003\ \mathrm{Mb}}$$

### Neoadjuvant therapy and treatment outcomes

Neoadjuvant regimens were administered as various combinations of systemic chemotherapy/targeted inhibitors according to the patient’s HER2 and HR status and the physician’s decision. Regimens included were taxanes (T), epirubicin (E), doxorubicin (A), cyclophosphamide (C), 5-fluorouracil (F), trastuzumab (T), pertuzumab (P), carboplatin (Cb), and capecitabine (X). All combination regimens were administered at standard dosing schedules. Pathological complete response (pCR) was assessed using the completely resected breast specimen and all regional lymph nodes sampled after neoadjuvant therapy. pCR was defined as the absence of residual invasive cancer cells on hematoxylin and eosin evaluation of all specimens.

### Survival analysis

Disease-free survival (DFS) was defined as the time in months from the date of diagnosis until the date of either disease relapse/disease progression or last follow-up. The proportion of patients who had radiologically confirmed disease relapse (PD ratio) was calculated as the ratio of patients with relapse to the total number of patients who remained disease-free at that particular year of follow-up, excluding the number of patients who were lost to follow-up. The data cut-off date was October 15, 2021.

### Statistical analysis

The R statistics package (R version 4.0.5; R: The R-Project for Statistical Computing, Vienna, Austria) was used to analyze the data. Patient characteristics and sequencing results were summarized with descriptive statistics. Mutation profiles of HER2-positive, HER2-low, and HER2-zero groups in the GDPH cohort were compared using Fisher’s exact test and Wilcoxon signed-rank test, as deemed appropriate. Data clustering was performed using non-negative matrix factorization (NMF) based on Euclidean distance. R package NMF was implemented to estimate the best rank using Lee’s algorithm with the following initial parameters: 2:8 for rank, and numeric random seed (seed = 123456). The most ideal values for cophenetic consensus were observed at ranks 3–4; hence, we selected rank 3 to cluster our data into three subgroups. Kaplan-Meier survival curves with log-rank statistics were plotted to compare DFS among subgroups. All statistical tests were performed as two-sided test and significance level was set at *P* < 0.05.

## Results

### Baseline characteristics of the cohort

**Fig. 1:**
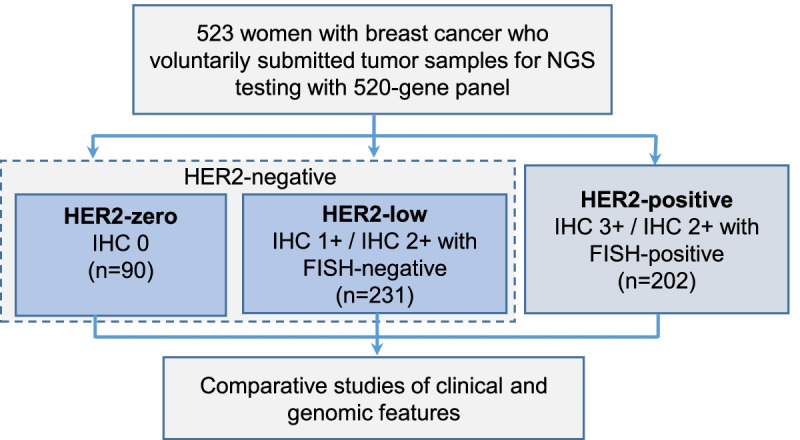
Diagram of the study design

Figure 1 illustrates our study design. This study included 523 women with breast cancer with a median age of 48 years (range 25–85 years). The majority of the cohort was comprised of patients with early-stage breast cancer (72.3%, *n* = 378), with invasive carcinoma of no special type (NST) (92.4%, *n* = 483), and HR-positive breast cancer (72.7%, *n* = 380). The cohort was further stratified according to HER2 status based on the 2018 ASCO/CAP guidelines. A total of 321 patients were HER2-negative as per the guidelines, with 90 patients (17.2%) having IHC 0, defined as HER2-zero, and 231 patients (44.2%) having either IHC 1+ or IHC 2+ with negative FISH results, defined as HER2-low. The remaining 202 patients (38.6%) had either IHC 3+ or IHC 2+ with positive FISH results, defined as HER2-positive. Table 1 summarizes the clinicopathological characteristics of the cohort and the patient subgroups. The patients stratified according to HER2 status had similar age, menopausal status, and pathological stage distribution, but had statistically different histological types, histological grades, hormone receptor (HR) status, Ki67 expression levels, and IHC-based molecular subgroup status.

**Table 1:** Baseline clinicopathological characteristics of the cohort

Clinicopathological characteristics	Total (*n*=523); *n*(%)	IHC/FISH-based HER2 status subgroup; n(%)			
		HER2-zero (*n*=90)	HER2-low (*n*=231)	HER2-positive (*n*=202)	*p*-value
Age (median [range], years)	48 [25-85]	46[25-85]	49[27-76]	49.5[27-74]	ns
Menopausal status					
Pre-menopause	297(56.8%)	56(62.2%)	131(56.7%)	110(54.5%)	ns
Post-menopause	226(43.2%)	34(37.8%)	100(43.3%)	92(45.5%)	
Tumor (T) stage					
T1-T2	469(89.7%)	80(88.9%)	208(90.0%)	181(89.6%)	ns
T3-T4	51(9.7%)	8(8.9%)	22(9.5%)	21(10.4%)	
Unknown	3(0.6%)	2(2.2%)	1(0.5%)	0	
Lymph node (N) stage					
N0-N1	400(76.5%)	68(75.6%)	176(76.2%)	156(77.2%)	ns
N2-N3	121(23.1%)	20(22.2%)	55(23.8%)	46(22.8%)	
Unknown	2(0.4%)	2(2.2%)	0	0	
Metastasis (M) stage					
M0	495(94.6%)	84(93.3%)	226(97.8%)	185(91.6%)	Zero vs Low, *p* =0.036
M1	26(5.0%)	4(4.4%)	5(2.2%)	17(8.4%)	Zero vs Positive, ns
Mx	2(0.4%)	2(2.2%)	0	0	Low vs Positive, *p* =0.004
Pathological stage					
I-II	378(72.3%)	65(72.2%)	168(72.7%)	145(71.8%)	ns
III-IV	142(27.1%)	23(25.6%)	62(26.8%)	57(28.2%)	
Unknown	3(0.6%)	2(2.2%)	1(0.5%)	0	
Histological grade					
1	13(2.5%)	3(3.3%)	8(3.5%)	2(1.0%)	Zero vs Low, *p* <0.001
2	248(47.4%)	31(34.4%)	137(59.3%)	80(39.6%)	Zero vs Positive, ns
3	248(47.4%)	50(55.6%)	82(35.5%)	116(57.4%)	Low vs Positive, *p* <0.001
Unknown	14(2.7%)	6(6.7%)	4(1.7%)	4(2.0%)	
Histological type					
Invasive carcinoma of no special type (NST)	483(92.4%)	77(85.6%)	214(92.6%)	192(95.0%)	Zero vs Low, *p* =0.043
Lobular, invasive	15(2.9%)	4(4.4%)	10(4.3%)	1(0.5%)	Zero vs Positive, *p* =0.008
Other invasive histology	25(4.7%)	9(10.0%)	7(3.1%)	9(4.5%)	Low vs Positive, *p* =0.023
Estrogen Receptor (ER) status					Zero vs Low, *p* <0.001
Positive	360(68.8%)	59(65.6%)	198(85.7%)	103(51.0%)	Zero vs Positive, *p* =0.022
Negative	163(31.2%)	31(34.4%)	33(14.3%)	99(49.0%)	Low vs Positive, *p* <0.001
Progesterone Receptor (PR) status					Zero vs Low, *p* <0.001
Positive	335(64.1%)	52(57.8%)	191(82.7%)	92(45.5%)	Zero vs Positive, ns
Negative	188(35.9%)	38(42.2%)	40(17.3%)	110(54.5%)	Low vs Positive, *p* <0.001
Hormone Receptor (HR) status					Zero vs Low, *p* <0.001
Positive	380(72.7%)	60(66.7%)	202(87.4%)	118(58.4%)	Zero vs Positive, ns
Negative	143(27.3%)	30(33.3%)	29(12.6%)	84(41.6%)	Low vs Positive, *p* <0.001
Ki67 expression level (median, IQR)	30.0%[15.0%, 50.0%]				
Ki67 expression status (cut-off 30%)					Zero vs Low, *p* =0.013
Low (<30%)	225(43.4%)	38(42.2%)	132(57.9%)	55(27.5%)	Zero vs Positive, *p* =0.015
High (≥30%)	293(56.6%)	52(57.8%)	96(42.1%)	145(72.5%)	Low vs Positive, *p* <0.001
Immunohistochemistry-based surrogate molecular subgrouping					
Luminal A (ER±/PR±/HER2-/Ki67<14%)	91(17.4%)	25(27.8%)	66(28.6%)	0	Zero vs Low, *p* <0.001
Luminal B HER2- (ER±/PR±/HER2-/Ki67≥14%)	171(32.7%)	35(38.9%)	136(58.9%)	0	Zero vs Positive, *p* <0.001
Luminal B HER2+ (ER±/PR±/HER2+/Ki67≥14%)	118(22.6%)	0	0	118(58.4%)	Low vs Positive, *p* <0.001
HER2-enriched (ER-/PR-/HER2+)	84(16.1%)	0	0	84(41.6%)	
Triple-negative (ER-/PR-/HER2-)	59(11.3%)	30(33.3%)	29(12.6%)	0	

*Abbreviations*: *ns* not statistically significant

### Distinct clinical features of HER2-low breast tumors

**Fig. 2:**
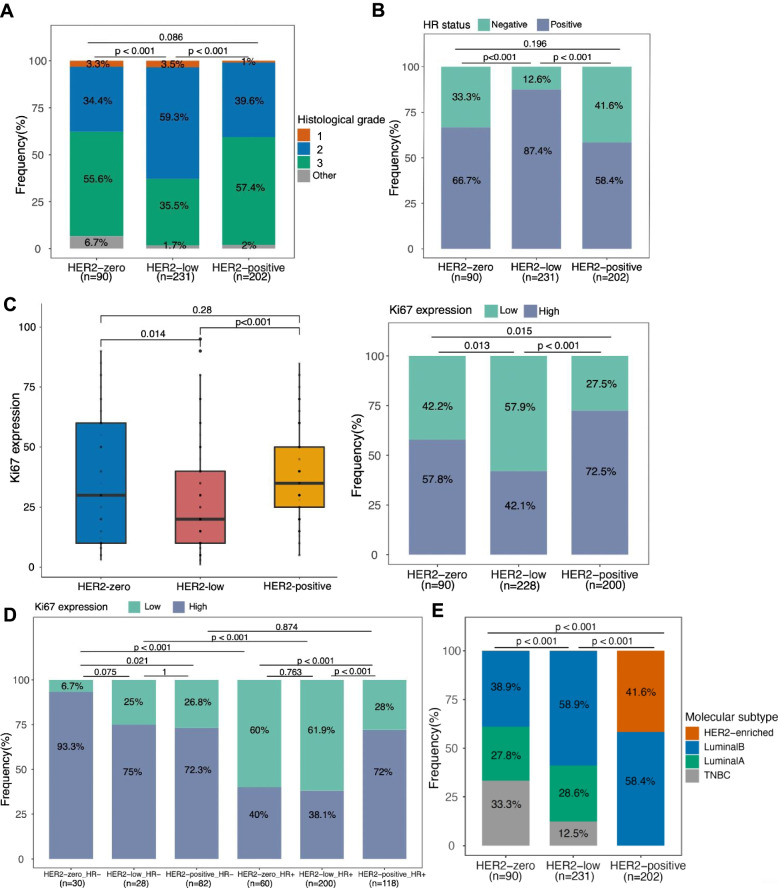
HER2 subgroups had different clinical features, including **A** histological grade, **B** hormone receptor (HR) expression status, **C,D** Ki-67 expression levels, and **E** IHC-based molecular subtype distribution

The HER2-low subgroup were distinctive based on three criteria: (1) the subgroup exhibited more histological grade 2 than HER2-zero (59.3% vs. 34.4%, *p* < 0.001) and more than HER2-positive (59.3% vs. 39.6%, *p* < 0.001) (Fig. 2A); (2) The HER2-low subgroup had significantly more patients with HR-positive status than HER2-zero patients (87.4% vs. 66.7%, *p* < 0.001) and HER2-positive patients (87.4% vs. 41.6%, *p* < 0.001) subgroups (Fig. 2B); and (3) the HER2-low subgroup had significantly lower Ki67 expression levels than the HER2-zero patients (*p* = 0.014) and the HER2-positive patients (*p* < 0.001)(Fig. 2C). The HER2-zero and HER2-positive subgroups had no statistical difference in histological grade (*p* = 0.086), HR status (*p* = 0.196), and Ki67 expression level (*p* = 0.28). We also used a median cut-off of 30% to stratify the Ki67 as low (<30%) and high (≥30%). Among the HR-negative tumors, the HER2-zero subgroup had a significantly higher proportion of tumors with high Ki67 expression than the HER2-positive subgroup (99.3% vs. 72.3%, *p* = 0.021). Among the HR-positive tumors, the HER2-positive subgroup had a significantly higher proportion of tumors with high Ki67 expression than HER2-low (72.0% vs. 38.1%, *p* < 0.001) and HER2-zero subgroup (72.0% vs. 40.0%, *p* < 0.001) (Fig. 2D). Moreover, among the HER2-low subgroup, those with HR-positive status had a significantly higher proportion of low Ki67 expression than those with HR-negative status (61.9% vs. 25.0%, *p* < 0.001) (Fig. 2D). According to IHC-based molecular subtype distribution, the HER2-low subgroup was comprised predominantly of luminal B tumors (58.9%) and was 28.6% of luminal A tumors, and 12.5% triple-negative breast cancer (TNBC). This distribution was significantly different from HER2-zero (*p* < 0.001) and HER2-positive (*p* < 0.001) subgroups (Fig. 2E). As compared with the HER2-zero subgroup, the HER2-low subgroup had a significantly higher proportion of luminal B tumors (58.9% vs. 38.9%, *p* = 0.002), and a significantly lower proportion of TNBC tumors (12.5% vs. 33.3%, *p* < 0.001). It should be noted that all luminal B tumors in HER2-low and HER2-zero subgroups were HER2-negative. Taken together, these findings indicate the difference in clinical features among HER2-zero, HER2-low, and HER2-positive breast tumors.

### Mutation landscape of the cohort

Genomic results from targeted sequencing of breast tumors using a large 520-gene panel were analyzed and are summarized in supplementary Additional file 1: Fig. S1. Tumor protein 53 (*TP53*) and phosphatidylinositol-4,5-bisphosphate 3-kinase catalytic subunit alpha (*PIK3CA*), respectively, were detected in 54% and 46% of the cohort and were the two most frequently mutated genes. Copy number amplifications in cyclin-dependent kinase 12 (*CDK12*) were only exclusively detected together with copy number amplifications in *HER2* (*n* = 113; *p* < 0.001; Additional file 1: Fig. S1).

### Distinct somatic mutation landscape and mutated pathways across HER2 subgroup

**Fig. 3:**
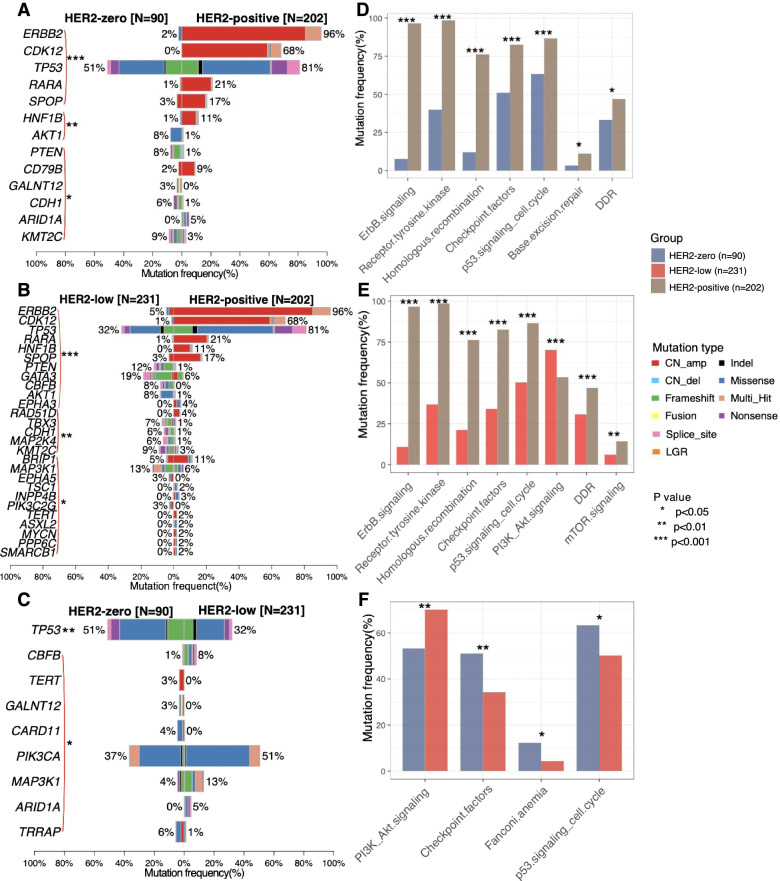
HER2 subgroups had differentially mutated genes (**A–C**) and molecular signaling pathways (**D–F**)

We then compared the somatic mutation landscape according to the HER2 subgroups. Besides *HER2* gene amplifications, HER2-positive breast tumors were detected with more copy number amplification in genes including *CDK12* (*p* < 0.001), retinoic acid receptor alpha (*RARA*; *p* < 0.001), and speckle-type POZ protein (*SPOP*; *p* < 0.001) and significantly higher *TP53* mutation rates (*p* < 0.001) as compared with HER2-zero (Fig. 3A, Additional file 1: Fig. S2A) and HER2-low breast tumors (Fig. 3B, Additional file 1: Fig. S2B). HER2-low breast tumors had significantly more frequent mutations in phosphatase and tensin homolog (*PTEN*; *p* < 0.001), GATA binding protein 3 (*GATA3*; *p* < 0.001), core-binding factor subunit beta (*CBFB*; *p* < 0.001), and AKT serine/threonine kinase 1 (*AKT1*; *p* < 0.001) than HER2-positive breast tumors (Fig. 3B, Additional file 1: Fig. S2B). HER2-low breast tumors also had significantly higher mutations rates in *CBFB* (*p* < 0.05), *PIK3CA* (*p* < 0.05), mitogen-activated protein kinase kinase kinase 1 (*MAP3K1*; *p* < 0.05), and AT-rich interaction domain 1A (*ARID1A*; *p* < 0.05) as compared with HER2-zero breast tumors (Fig. 3C, Additional file 1: Fig. S2C). Moreover, HER2-zero breast tumors had significantly higher mutation rates in *TP53* (*p* < 0.01), telomerase reverse transcriptase (*TERT*; *p* < 0.05), polypeptide N-acetylgalactosaminyltransferase 12 (*GALNT12*; *p* < 0.05), caspase recruitment domain family member 11 (*CARD11*; *p* < 0.05), and transformation/transcription domain-associated protein (*TRRAP*; *p* < 0.05) than HER2-low tumors (Fig. 3C, Additional file 1: Fig. S2C).

Of the *TP53* mutations detected, approximately 80% were located across exons 5–8 for all HER2 subgroups (Additional file 1: Fig. S3). Overall, the *TP53* exon 5–8 mutation rate was 40.0% (36/90) for HER2-zero, 26.0% (60/231) for HER2-low, and 67.8% (137/202) for HER2-positive breast tumors. *PIK3CA* mutations were distributed across the functional domains (Additional file 1: Fig. S4). *PIK3CA* E545K (*n* = 11) and H1047R/L (*n* = 11) were the most common *PIK3CA* mutations in HER2-zero. *PIK3CA* H1047R/L was the most common in HER2-low (*n* = 71) and HER2-positive (*n* = 46) breast tumors.

We also explored the genes and signaling pathways commonly mutated in HER2 subgroups and analyzed them according to HR status and IHC-based molecular subtypes. The two commonly mutated genes across HER2 subgroups and HR status were *TP53* and *PIK3CA* (Additional file 1: Fig. S5A). No genes or signaling pathways were differentially mutated between HER2-low and HER2-zero tumors with HR-negative status (Additional file 1: Fig. S5B). Only the mutation rate of RPTOR-independent companion of mammalian target of rapamycin (RICTOR) was found to be significantly higher in HER2-zero as compared to HER2-low tumors with HR-positive status (7% [4/60] vs. 0% [0/220], *p* = 0.011; Additional file 1: Fig. S5C). Despite being numerically higher in HER2-zero subgroup, *TP53* mutation rate was not statistically different between HER2-zero and HER2-low breast tumors regardless of HR status (HR-positive [HER2-zero vs. HER2-low], 33.3% vs. 26.2%, *p* = 0.325; HR-negative 86.7% vs. 72.4%, *p* = 0.209; Additional file 1: Fig. S6A). Likewise, *PIK3CA* mutations were numerically higher in HER2-low subgroup; however, no statistical difference was observed between HER2-low and HER2-zero subgroups with HR-negative (23.3% vs. 37.9%, *p* = 0.267) and HR-positive status (43.3% vs. 52.5%, *p* = 0.241) (Additional file 1: Fig. S6B). However, as compared with HR-negative breast tumors, those with HR-positive status had significantly fewer *TP53* mutations among HER2-zero (33.3% [20/60] vs. 86.7% [26/30], *p* < 0.001) and HER2-low (26.2% [53/202] vs. 72.4% [21/29], *p* < 0.001), but not among HER2-positive breast tumors (79.7% [94/118] vs. 85.7% [72/84], *p* = 0.351) (Additional file 1: Fig. S6A). *PIK3CA* and *TP53* were the two commonly mutated genes in HER2-low and HER2-zero tumors across IHC-based molecular subgrouping (Additional file 1: Fig. S7). *TP53* mutation rates were numerically higher in HER2-zero tumors but not statistically different between HER2-zero and HER2-low tumors with TNBC (86.7% vs. 72.4%, *p* = 0.209), luminal A (16.0% vs. 12.1%, *p* = 0.730), and luminal B (45.7% vs. 33.1%, *p* = 0.172; Additional file 1: Fig. S8A). According to IHC-based molecular subgrouping, *TP53* mutations were more common in TNBC and less frequent in luminal A for both HER2-zero and HER2-low tumors (Additional file 1: Fig. S8A). *PIK3CA* mutation rates were numerically higher in HER2-low tumors but not statistically different between HER2-low and HER2-zero tumors with TNBC (37.9% vs. 23.3%, *p* = 0.267), luminal A (63.3% vs. 56.0%, *p* = 0.630), and luminal B (47.1% vs. 34.3%, *p* = 0.118) (Additional file 1: Fig. S8A). *PIK3CA* mutations were more frequent in luminal A and least common in TNBC (Additional file 1: Fig S8B). No genes or signaling pathways were differentially mutated between TNBC tumors from HER2-low and HER2-zero subgroups (Additional file 1: Fig. S9A). The lack of statistical significance in these subgroup analyses might be due to the smaller sample size in each subgroup after stratification. In luminal A tumors, the mutation rate of spliceosome factor 3b (SF3B1) was found to be significantly higher for HER2-zero than HER2-low subgroup (12% [3/25] vs. 0% [0/66], *p* < 0.05; Additional file 1: Fig. S9B). In luminal B tumors, the mutations rates of *TERT* (6% [2/35] vs. 0% [0/136], *p* < 0.05), *GALNT12* (6% [2/35] vs. 0% [0/136], *p* < 0.05), and *RICTOR* (11% [3/35] vs. 0% [0/136], *p* < 0.05) were significantly higher for HER2-zero than HER2-low subgroup (Additional file 1: Fig. S9C).

We further investigated the signaling pathways commonly mutated in each HER2 subgroup. Aside from the ErbB/HER pathway, HER2-positive breast tumors had frequent mutations in genes involved in receptor tyrosine kinase signaling (*p* < 0.001), homologous recombination (*p* < 0.001), checkpoint factors (*p* < 0.01), and p53 signaling and cell cycle (*p* < 0.01) as compared with HER2-zero (Fig. 3D) and HER2-low breast tumors (Fig. 3E). In contrast, HER2-low tumors had significantly more mutations involved in PI3K-Akt signaling pathways as compared with HER2-positive (*p* < 0.001) (Fig. 3E) and HER2-zero breast tumors (*p* < 0.01) (Fig. 3F). HER2-zero tumors had significantly more mutations in checkpoint factors (*p* < 0.01), Fanconi anemia (*p* < 0.05), and p53 signaling and cell cycle pathway (*p* < 0.05) than HER2-low breast tumors (Fig. 3F).

Although mutation detection rates were varied across the HER2 subgroups, the comparison of their TMB demonstrated no statistical difference (Additional file 1: Fig. S10). The median TMB was 3.67 (range 0–32.11) mutations/Mb for HER2-positive, 3.67 (range 0–19.27) mutations/Mb for HER2-low, and 2.75 (range 0–32.11) mutations/Mb for HER2-zero breast tumor.

Taken together, these data suggest some significant differences in the genomic features for breast tumors with HER2-positive, HER2-low, and HER2-zero status.

### Mutations in HER family among the HER2 subgroups

We also explored the mutation frequency of other members of the ErbB/HER family. The HER family consists of four receptor tyrosine kinases, epidermal growth factor receptor (*EGFR* or *ERBB1*), *ERBB2 (*or *HER2)*, *ERBB3*, and *ERBB4*. Additional file 1: Table S1 summarizes the distribution of HER family mutations of the cohort. HER2-positive breast tumors had the highest mutation rate in genes in the HER family (11.9%) but were not statistically different from the mutation rate in HER2-low (8.7%) and HER2-zero breast tumors (7.8%) (Additional file 1: Fig. S11A). SNVs affecting the *ERBB2* gene, such as missense mutations and splice-site mutations, were detected in two HER2-zero patients (Additional file 1: Fig. S11B), four HER2-low patients (Additional file 1: Fig. S11C), and 13 HER2-positive patients (Additional file 1: Fig. S11D). Concurrent *ERBB2* amplification and *ERBB2* P122A and D277E missense mutations were detected in two patients with IHC 3+. *EGFR* mutations were more frequently detected in HER2-positive tumors (4.9%). *ERBB3* mutations were detected in four patients (4.4%) with HER2-zero tumors. *ERBB4* mutations were detected in four patients each with HER2-low (1.7%) and HER2-positive tumors (2.0%).

### Clustering analysis reveals distinct mutational features and pathways

We then performed data clustering using the NMF approach to identify common clusters of mutations from the mutational profile of the cohort. *HER2* gene amplification was excluded for this analysis to eliminate the confounding effects of the HER2 subgrouping.

**Fig. 4:**
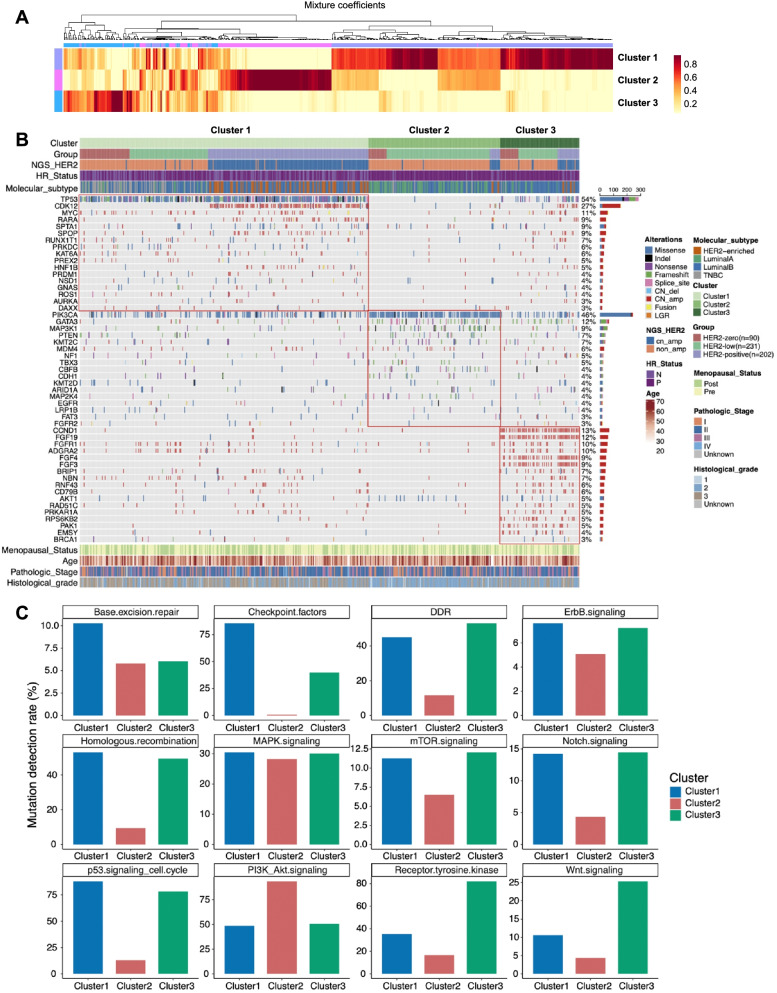
Distinct mutational features as demonstrated by clustering of mutation profiles of the cohort. **A** Heat map illustrating the unsupervised hierarchical clustering of mutation profile of the cohort. **B** Oncoprint summarizing the mutational features of each cluster. **C** Comparison of the molecular signaling pathways that are differentially mutated in each cluster

Unsupervised clustering of the mutation profile of the cohort revealed three molecularly distinct clusters as shown by the heat map in Fig. 4A and the oncoprint in Fig. 4B. Cluster 1 was enriched in *TP53* mutations, *CDK12*, *MYC*, and *RARA* amplifications. Cluster 2 was enriched in *PIK3CA*, *GATA3*, and *MAP3K1* mutations, with a lesser number of gene amplification and the absence of *TP53* mutation. Cluster 3 was enriched in copy number amplifications of various genes, including cyclin D1 (*CCND1*), fibroblast growth factor (*FGF*) 3 (*FGF3*), *FGF4*, *FGF19*, ribosomal protein S6 kinase B2 (*RPS6KB2*), P21 activated kinase 1 (*PAK1*), and EMSY BRCA2-interacting transcriptional repressor (*EMSY*), which are associated with chromosome 11q13 loci amplification, as well as FGF receptor 1 (*FGFR1*) and adhesion G protein-coupled receptor A2 (*ADGRA2*), which are both located in chromosome 8p12.

Pathway analysis demonstrated that genes in Cluster 1 were involved in base excision repair, homologous recombination, p53 signaling and cell cycle, checkpoint factor, ErbB signaling, and Notch signaling. Cluster 2 was comprised of genes involved in PI3K/Akt pathway. Cluster 3 was comprised of genes involved in DNA damage response, receptor tyrosine kinase, and Wnt signaling pathway (Fig. 4C).

According to HER2 subgrouping, Cluster 1 comprised of 55.6% (168/302) HER2-positive, 27.2%% (82/302) HER2-low, and 17.2% (52/302) HER2-zero breast tumors. Of them, 178 patients (58.9%) were HR-positive. Cluster 2 comprised of 78.3% (108/138) HER2-low, 13.7% (19/139) HER2-zero, and 8.0% (11/138) HER2-positive breast tumors. Cluster 3 comprised of 49.4% (41/83) HER2-low, 27.7% (23/83) HER2-positive, and 22.9% (19/83) HER2-zero breast tumors. A majority of the patients in Cluster 2 (92.8%, 128/138) and Cluster 3 (89.2%, 74/83) were HR-positive (Additional file 1: Fig. S12A). According to the IHC-based molecular subgrouping, Cluster 1 was comprised of 54.0% (163/302) luminal B, 24.5% (74/302) HER2-enriched, 16.6% (50/302) TNBC, and 5% (15/302) luminal A. Cluster 2 was comprised of 47.1% (65/138) luminal A, 45.7% (63/138) of luminal B, 4.3% (6/138) TNBC, and 2.9% (4/138) HER2-enriched. Cluster 3 was comprised of a majority of luminal B (75.9%, 63/83), 13.3% (11/83) luminal A, 7.2% (6/83) HER2-enriched, and 3.6% (3/83) TNBC (Additional file 1: Fig. S12B).

These findings suggest the existence of molecular subsets within HER2-positive, HER2-low, and HER2-zero subgroups harboring certain mutational features that could contribute to the molecular heterogeneity of these tumors.

### Clinical outcomes

**Fig. 5:**
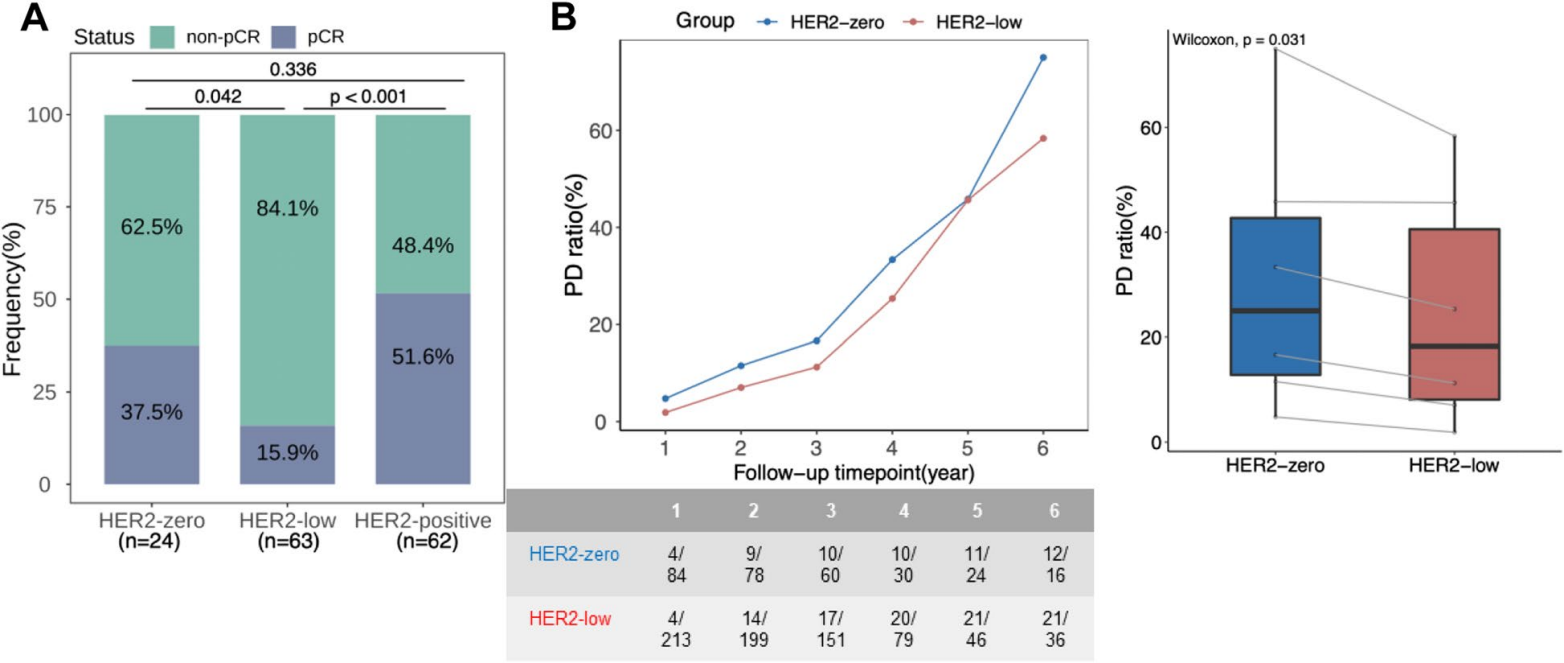
HER2-low breast tumor had different clinical outcomes than HER2-zero. **A** HER2-low subgroup had lower pathological complete response (pCR) rates. **B** Proportion of relapsed/progressed patients were fewer in HER2-low subgroup across follow-up time points. The table below summarizes the number of relapsed patients over the total number of included patients per time point (in year) per subgroup. The gray connecting lines in the box plot denote the follow-up data per year corresponding to the data point in the dot plot on the left

We further analyzed the treatment outcomes of 149 patients who received neoadjuvant therapy with available pathological response data. HER2-low subgroup had significantly lower pCR rates than HER2-zero (15.9% [10/63] vs. 37.5% [9/24], *p* = 0.042) and HER2-positive subgroups (15.9% [10/63] vs. 51.6% [32/62], *p* < 0.001) (Fig. 5A). pCR rates were numerically lower in HER2-low subgroup with HR-positive (9.3% vs. 20.0%, *p* = 0.358), HR-negative (55.6% vs. 66.7%, *p* = 1), TNBC (55.6% vs. 66.7%, *p* = 1), and luminal B (10.8% vs. 25.0%, *p* = 0.340), but was numerically higher in luminal A tumors (5.9% vs. 0.0%, *p* = 1); however, statistical analysis showed no significant difference between HER2-low and HER2-zero subgroups across HR status and IHC-based molecular subtypes possibly due to lower sample size in some subgroups (Additional file 1: Fig. S13).

We also analyzed the DFS for all patients. DFS were comparable between HER2-low and HER2-zero subgroups (*p* = 0.271; Additional file 1: Fig. S14A), regardless of pCR status (Additional file 1: Fig. S15), HR status (Additional file 1: Fig. S16), and IHC-based molecular subtypes (Additional file 1: Fig. S17). It should be noted that the DFS data was only 10.1% (53/523) mature and results might be inconclusive. Hence, we performed further analysis to compare the DFS of patients who experienced disease relapse (*n* = 53) according to HER2 subgroups. As expected, women with HER2-zero (*n* = 12; *p* = 0.025) and HER2-low (*n* = 21; *p* = 0.031) tumors had significantly shorter DFS than HER2-positive tumors (*n* = 20); however, DFS was comparable between HER2-zero and HER2-low subgroups (Additional file 1: Fig. S14B). Interestingly, the proportion of relapsed/progressed patients was significantly lower in HER2-low subgroup than HER2-zero subgroup across follow-up time points (*p* = 0.031; Fig. 5B). Despite comparable DFS, these findings suggest that HER2-low tumors had different clinical outcomes than HER2-zero tumors as shown by the significantly lower pCR rate but a higher proportion of disease-free patients across follow-up time points.

## Discussion

In this study, we included a cohort of 523 Chinese women with breast cancer stratified into three subgroups according to their HER2-expression status as HER2-positive, HER2-low, and HER2-zero to compare their clinicopathological characteristics and NGS-based genetic alteration landscape. Our results describe the differences among the three HER2 categories at the clinicopathological and molecular level. From a clinical perspective, HER2-low patients were significantly more likely to be HR positive, with significantly lower Ki-67 expression level, and had significantly lower pCR rate with neoadjuvant chemotherapy than HER2-zero patients. From a molecular perspective, HER2-low tumors displayed a different somatic mutation landscape and mutated pathways, such as the higher prevalence of *PIK3CA* mutations and lower prevalence of *TP53* mutations than HER2-zero tumors. However, whether these differences in terms of molecular features are at least partly driven by the distinct clinicopathological features remains to be established.

In recent years, anti-HER2 treatment has successfully expanded its clinical application to target HER2-low expressing tumors using novel ADCs such as T-DXd and SYD 985. These novel ADCs were designed to effectively be anchored and delivered into the cell even those with low HER2 expression level to exert their cytotoxic effects via by-stander effect with significantly enhanced potency compared to the previous generation of ADCs [25, 33]. Since HER2-low expressing breast tumors constitute nearly half of all breast tumors, the recent availability of an established therapeutic strategy with novel ADCs for this subgroup have triggered new investigations to comprehensively characterize the clinical and genetic features of this new patient subgroup. Recently, Denkert et al. conducted a study by pooling individual patient data out of 2310 patients with HER2-negative primary breast cancer defined by the ASCO/CAP algorithm from four prospective neoadjuvant clinical trials [21]. Their findings, in the setting of clinical trial cohorts, revealed the distinct clinical features of HER2-low-positive breast cancer, such as significantly higher HR-positive expression, lower Ki-67 expression, and lower complete pathological response to neoadjuvant chemotherapy but longer overall survival, as compared with HER2-zero breast cancer. Moreover, they also found that HER2-low-positive breast tumors had a higher prevalence of *PIK3CA* mutations and a lower prevalence of *TP53* mutations than HER2-zero breast tumors. Their study provided evidence to consider HER2-low tumors as a new subtype of breast cancer with distinct biology, clinical characteristics, and prognosis from HER2-zero tumors. Our findings, while in the real-world patient setting, are consistent with the findings reported by Denkert et al [21]. Findings from both cohorts similarly revealed that HER2-low breast tumors had higher frequency of *PIK3CA* mutations, lower frequency of *TP53* mutations, and lower pCR rates with neoadjuvant therapy as compared with HER2-zero breast tumors.

In our study, the lower pCR rates of HER2-low subgroup had a similar trend in HR-positive, HR-negative/TNBC, and luminal B tumors; however, these comparisons across HR status and IHC-based molecular subtypes were inconclusive due to the small sample size of some of the subgroups (i.e., HR-negative and TNBC only had nine patients each per subgroup, luminal A only had three patients in HER2-zero and 17 in HER2-low, and luminal B only had 12 and 37 patients). Despite the inconclusive DFS due to the largely immature survival data from our cohort, our findings still showed that a significantly lesser proportion of women with HER2-low breast tumors experienced disease relapse up to 6 years of follow-up. This finding suggest that women with HER2-low are expected to have longer DFS and better survival outcomes than HER2-zero breast tumors.

The observed differences in clinical outcomes regarding pCR rates and survival trends could be attributed to differences in IHC-based molecular subtype distribution and molecular features, such as more luminal B and fewer TNBC subtypes, less *TP53* mutations but more *PIK3CA* mutation in the HER2-low tumors than in the HER2-zero tumors. The enriched mutations in genes involved in the PI3K-Akt pathway suggest a potential therapeutic strategy of combining chemotherapy and PI3K-related targeted therapy to improve the pCR rate in women with HER2-low tumors, which have been successfully demonstrated in various clinical trials [34–36]. It might also be possible to add these inhibitors to ADC when treating the HER2-low patients with metastatic disease to enhance the overall response rates, which is 40% with T-DXd and 28% with SYD985 [23, 24, 33, 37]. Nonetheless, these speculations are only hypothesis-generating and needs further confirmation by rigidly designed clinical trials.

Consistent with our previous study and other existing reports [32, 38, 39], somatic mutational profiling using a 520-gene panel NGS showed that HER2-positive breast tumors had more frequent *TP53* mutations, *CDK12* amplifications, and *RARA* amplifications. It should be noted that in our cohort, 81% of HER2-positive tumors were detected with *TP53* mutations while all *CDK12* amplifications were concurrently detected with *HER2* amplification. *TP53* mutations, particularly in the DNA-binding domain spanning exons 5–8, results in the malfunctioning of DNA damage repair pathways and are the most frequently reported mutation in solid tumors, including breast cancer [29, 40]. *CDK12* is located in chromosome 17q12, which is a neighboring gene of HER2, and hence is frequently amplified with *HER2* [41, 42]. CDK12 regulates the alternative splicing of the genes involved in DNA damage response pathway and the pathogenesis of breast cancer [42, 43]. Consistently, our results demonstrate that the DNA damage response pathway and homologous recombination repair pathway were significantly altered with a higher frequency among HER2-positive breast tumors. In addition, our study showed that HER2-positive tumors also harbored the most frequent HER family genes mutations than the other two subgroups.

Our findings from the molecular data clustering showed that our breast cancer cohort can be classified into three clusters of differential gene mutation. Cluster 1 was enriched in *TP53* mutations. Cluster 2 was enriched in *PIK3CA* mutation but lack *TP53* mutation. Cluster 3 was enriched in copy number amplifications of various genes associated with the chromosome 11q13 loci, wherein coamplification of *CCND1* and *FGF3/4/19* was the most common. Chromosome 11q13 locus is considered gene-rich and is one of the most commonly aberrant chromosome loci in breast cancer [44]. Amplification of chromosome 11q13 has been reported in up to 15% of patients with breast cancer and is associated with endocrine therapy resistance and poor prognosis, particularly for ER-positive and invasive lobular carcinomas [44–46]. *CCND1* amplification has been associated with immunosuppression of the tumor microenvironment and poor outcomes with immune checkpoint inhibitors [47]. Furthermore, the amplification of chromosome 11q13 locus is commonly observed to be associated with amplifications at chromosome 8p12, particularly *FGFR1*, and appear to be mutually exclusive from *HER2* amplification [48].

Our study has some inherent limitations. Our study was carried out in a single institution, which could introduce patient selection bias. Our study only included tumor samples sequenced using a 520-gene panel, which might introduce patient sampling bias, such as the higher HER2 positive rate of our cohort despite being unselected for clinical or pathological features (~38.6%). However, a study with a larger cohort of unselected invasive breast cancer (*n* = 12,467) have reported an average HER2-positive rate of 24.7%, ranging 13.7 to 35.7% across 19 different institutions from China [4]. Hence, we consider that the HER2-positive rate of our cohort is within the acceptable range, albeit being at the higher end of the limit. Since both the HER2-low and HER2-zero subgroups were classified as HER2-negative, our analysis was unaffected by the HER2-positive rate except for a smaller subgroup when analyzing HR status and IHC-based molecular subtypes. Since the study was not selected for clinicopathological features, our cohort did not include an adequate sample size to perform subgroup matching based on HR or IHC-based molecular subtype, which limited our findings to determine whether the difference in molecular features observed for HER2-low tumors were driven by differences in clinicopathological features. Moreover, only the genomic profiles of the breast tumors were analyzed and lacked other omics data such as gene expression profiling or protein expression, which limits our conclusions on the signaling pathways involved in various HER2 subgroups. Our cohort lacked treatment outcomes for HER2-antibody-drug conjugates, PI3K inhibitors, and immune checkpoint inhibitors. Due to the retrospective nature of our study, the treatment and survival data of our cohort were incomplete, with some patients lost to follow-up. The survival data of this cohort remains immature (approximately 10% maturity); hence, a longer follow-up period is necessary for definitive analysis. Nevertheless, our observations suggest that HER2 low breast tumors are clinically and molecularly different from HER2-zero tumors. Our findings are hypothesis-generating. Further investigations are warranted to elucidate and establish the distinct features of HER2 low breast tumors.

## Conclusion

Our study demonstrates the complex genetic heterogeneity across breast tumors with varied HER2 status and identifies potential oncogenic mechanism that drives HER2-low breast tumor. Our findings indicate that patients with either IHC 1+ or IHC 2+ with FISH-negative results have distinct clinical and mutational features as compared with HER2-zero and HER2-positive breast tumors. Moreover, the accumulating evidence that HER2-low is clinically and biologically distinct from HER2-zero raises the need to explore diagnostic techniques and personalized therapeutic approaches that could identify and potentially benefit the subset of patients with HER2-low breast cancer.

## Supplementary Information

**Additional file 1: Table S1.** Mutations in other members of the *HER* family detected from our cohort. **Figure S1.** Somatic mutation landscape of the cohort. **Figure S2.** Comparison of mutational profile of the HER2 subgroups. **Figure S3.** Distribution of *TP53* mutations among the HER2 subgroups. **Figure S4.** Distribution of *PIK3CA* mutations among the HER2 subgroups. **Figure S5.** Genes frequently mutated across HER2 subgroups according to hormone receptor (HR) status. **Figure S6.** Comparison of *TP53* (**A**) and *PIK3CA* (**B**) mutation rates across HER2 subgroups according to hormone receptor (HR) status. **Figure S7.** Genes frequently mutated across immunohistochemistry-based molecular subgrouping between HER2-low and HER2-zero subgroups. TNBC, triple negative breast cancer. **Figure S8.** Comparison of *TP53* (**A**) and *PIK3CA* (**B**) mutation rates between HER2-zero and HER2-low subgroups according to immunohistochemistry-based molecular subgrouping. **Figure S9.** Comparison of genes (top 11) and signaling pathways (top 5) differentially mutated between HER2-low and HER2-zero subgroups according to immunohistochemistry-based molecular subgrouping: **A**. triple negative breast cancer (TNBC), **B**. luminal A, and **C**. luminal B. **Figure S10.** Tumor mutational burden (TMB) were comparable across HER2 subgroups. TMB were not statistically different among patients with HER2-zero (IHC 0), HER2-low (IHC1+ or IHC 2+/FISH-negative), and HER2-positive (IHC 3+ or IHC 2+/FISH-positive) breast tumors. **Figure S11.** Distribution of ERBB/HER family mutations according to HER2 status. **Figure S12.** Molecular clustering across hormone receptor (HR) status (**A**) and immunohistochemistry-based molecular subgrouping (**B**). **Figure S13.** Pathological complete response (pCR) rate is comparable between HER2-low and HER2-zero subgroups across hormone receptor (HR) status (**A-B**) and immunohistochemistry-based molecular subgrouping (**C-E**). **Figure S14.** Disease-free survival (DFS) was comparable between women with HER2-low and HER2-zero tumors as shown by Kaplan-Meier survival plot of the whole cohort (**A**) and when considering only the relapsed patients per group (**B**). **Figure S15.** Disease-free survival (DFS) was comparable between HER2-zero and HER2-low subgroups regardless of pathological complete response (pCR) status. **Figure S16.** Disease-free survival (DFS) was comparable between HER2-zero and HER2-low subgroups regardless of hormone receptor (HR) status. **Figure S17.** Disease-free survival (DFS) was comparable between HER2-zero and HER2-low subgroups across immunohistochemistry-based molecular subgrouping.

## Data Availability

The datasets analyzed for this study are available in the National Omics Data Encyclopedia (NODE), hosted by the Bio-Med Big Data Center (BMDC) available as accession number OEP001295.

## Abbreviations

ADC: Antibody drug conjugate
ASCO: American Society of Clinical Oncology
CAP: College of American Pathologists
CEP17: Chromosome 17 centromere
CNVs: Copy number variations
DFS: Disease-free survival
ER: Estrogen receptor
ERBB2: Erb-B2 receptor tyrosine kinase 2
FFPE: Formalin-fixed paraffin-embedded
FISH: Fluorescence in situ hybridization
GDPH: Guangdong Provincial People’s Hospital
HER2: Human epidermal growth factor receptor 2
HR: Hormone receptor
IHC: Immunohistochemistry
Indels: Insertion-deletions
NGS: Next-generation sequencing
NMF: Non-negative matrix factorization
pCR: Pathological complete response
PD: Progressive disease
PR: Progesterone receptor
SNP: Single-nucleotide polymorphism
SNVs: Single-nucleotide variations
SVs: Structural variations
TKI: Tyrosine kinase inhibitor
TMB: Tumor mutation burden
