# Supplementary material for: Distinct clinical and somatic mutational features of breast tumors with high-, low-, or non-expressing human epidermal growth factor receptor 2 status
Source: BMC Med. 2022 Apr 29;20:142. doi: 10.1186/s12916-022-02346-9 (PMC9052533; doi:10.1186/s12916-022-02346-9)
Supplement: Supplementary file 1 — Additional file 1: Table S1. Mutations in other members of the HER family detected from our cohort. Figure S1. Somatic mutation landscape of the cohort. Figure S2. Comparison of mutational profile of the HER2 subgroups. Figure S3. Distribution of TP53 mutations among the HER2 subgroups. Figure S4. Distribution of PIK3CA mutations among the HER2 subgroups. Figure S5. Genes frequently mutated across HER2 subgroups according to hormone receptor (HR) status. Figure S6. Comparison of TP53 (A) and PIK3CA (B) mutation rates across HER2 subgroups according to hormone receptor (HR) status. Figure S7. Genes frequently mutated across immunohistochemistry-based molecular subgrouping between HER2-low and HER2-zero subgroups. TNBC, triple negative breast cancer. Figure S8. Comparison of TP53 (A) and PIK3CA (B) mutation rates between HER2-zero and HER2-low subgroups according to immunohistochemistry-based molecular subgrouping. Figure S9. Comparison of genes (top 11) and signaling pathways (top 5) differentially mutated between HER2-low and HER2-zero subgroups according to immunohistochemistry-based molecular subgrouping: A. triple negative breast cancer (TNBC), B. luminal A, and C. luminal B. Figure S10. Tumor mutational burden (TMB) were comparable across HER2 subgroups. TMB were not statistically different among patients with HER2-zero (IHC 0), HER2-low (IHC1+ or IHC 2+/FISH-negative), and HER2-positive (IHC 3+ or IHC 2+/FISH-positive) breast tumors. Figure S11. Distribution of ERBB/HER family mutations according to HER2 status. Figure S12. Molecular clustering across hormone receptor (HR) status (A) and immunohistochemistry-based molecular subgrouping (B). Figure S13. Pathological complete response (pCR) rate is comparable between HER2-low and HER2-zero subgroups across hormone receptor (HR) status (A-B) and immunohistochemistry-based molecular subgrouping (C-E). Figure S14. Disease-free survival (DFS) was comparable between women with HER2-low and HER2-zero tumors as shown by [file 12916_2022_2346_MOESM1_ESM.pdf]

Supplementary Materials for

Distinct clinical and somatic mutational features of breast tumors with high-, low-, or non-expressing human epidermal growth factor receptor 2 status

Guochun Zhang, Chongyang Ren, Cheukfai Li, Yulei Wang, Bo Chen, Lingzhu Wen, Minghan Jia, Kai Li, Hsiaopei Mok, Li Cao, Xiaoqing Chen, Jiali Lin, Guangnan Wei, Yingzhi Li, Yuchen Zhang, Charles M. Balch, Ning Liao*

*** Corresponding Author:**

Ning Liao, M.D., Ph.D.

Department of Breast Surgery

Guangdong Provincial People's Hospital & Guangdong Academy of Medical Sciences

106 Zhongshan Er Road, Guangzhou, China 510080

Tel: (+86) 20-83827812-50910

Email address: syliaoning@scut.edu.cn

Supplementary data: 1 table, 17 figures

Table S1. Mutations in other members of the *HER* family detected from our cohort

Genes in the HER family	**HER2-zero** (n=90)	**HER2-low** (n=231)	**HER2-positive** (n=202)
EGFR	1 (1.1%)	8 (3.5%)	10 (4.9%)
ERBB2 (missense mutations)	2 (2.2%)	4 (1.7%)	11 (5.4%)
ERBB3	4 (4.4%)	5 (2.2%)	2 (1.0%)
ERBB4	0	4 (1.7%)	4 (2.0%)
All *HER* family mutations	7 (7.8%)	20 (8.7%)	24 (11.9%)

Abbreviations: EGFR, epidermal growth factor receptor; ERBB2, see HER2; ERBB3, Erb-B2 receptor tyrosine kinase 3; ERBB4, Erb-B2 receptor tyrosine kinase 4; FISH, fluorescence in situ hybridization; HER2, human epidermal growth factor receptor 2; IHC, immunohistochemistry

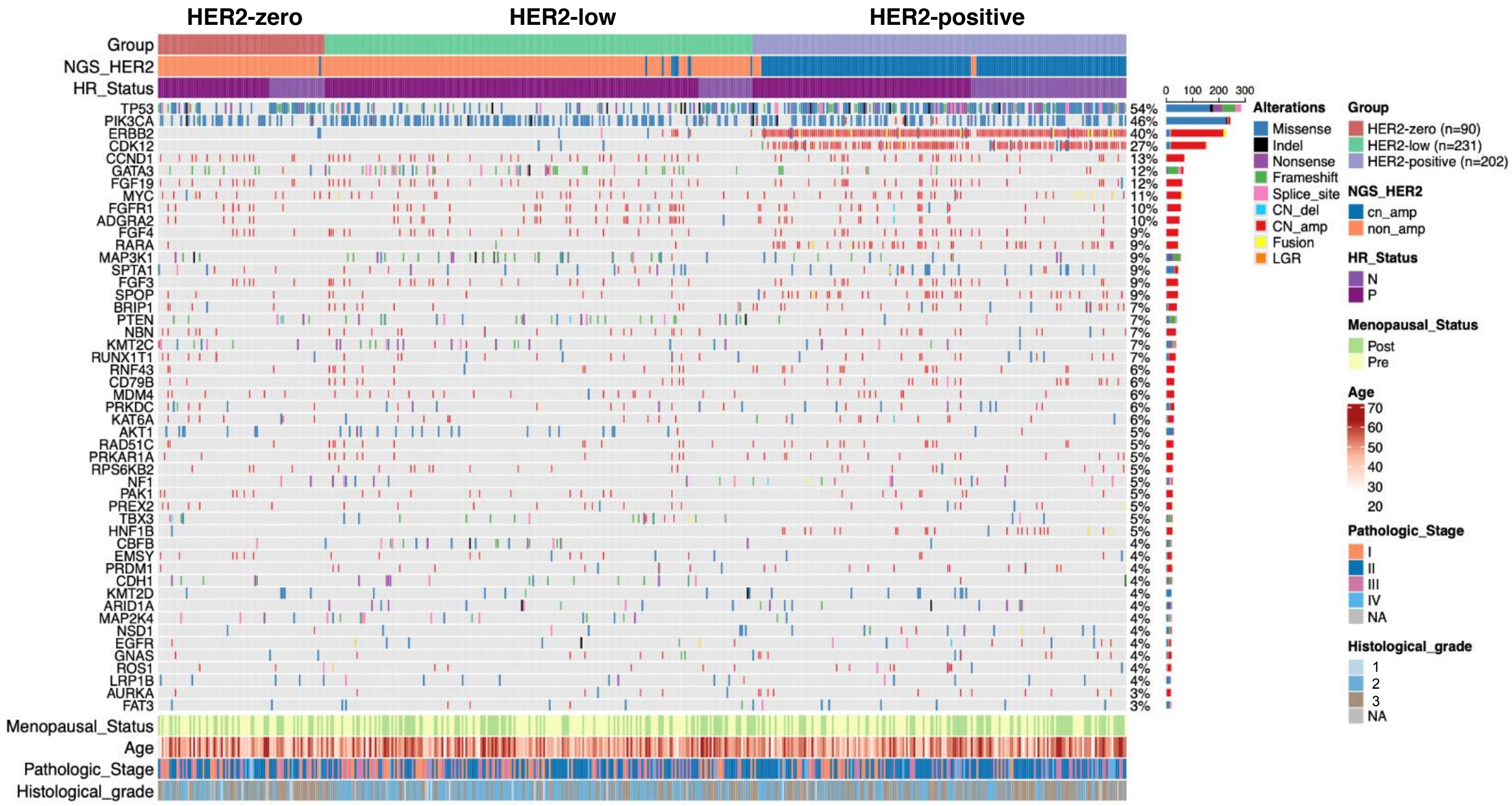

Figure S1. Somatic mutation landscape of the cohort. Oncoprint summarizing the mutated genes (right) and mutations types (colors) per patient arranged according to IHC/FISH subgroup as indicated at the top. Different colors at the top and bottom of the oncoprint represents other clinicopathological information of the patient including hormone receptor (HR) status, menopausal status, age, pathological stage, and histological stage.

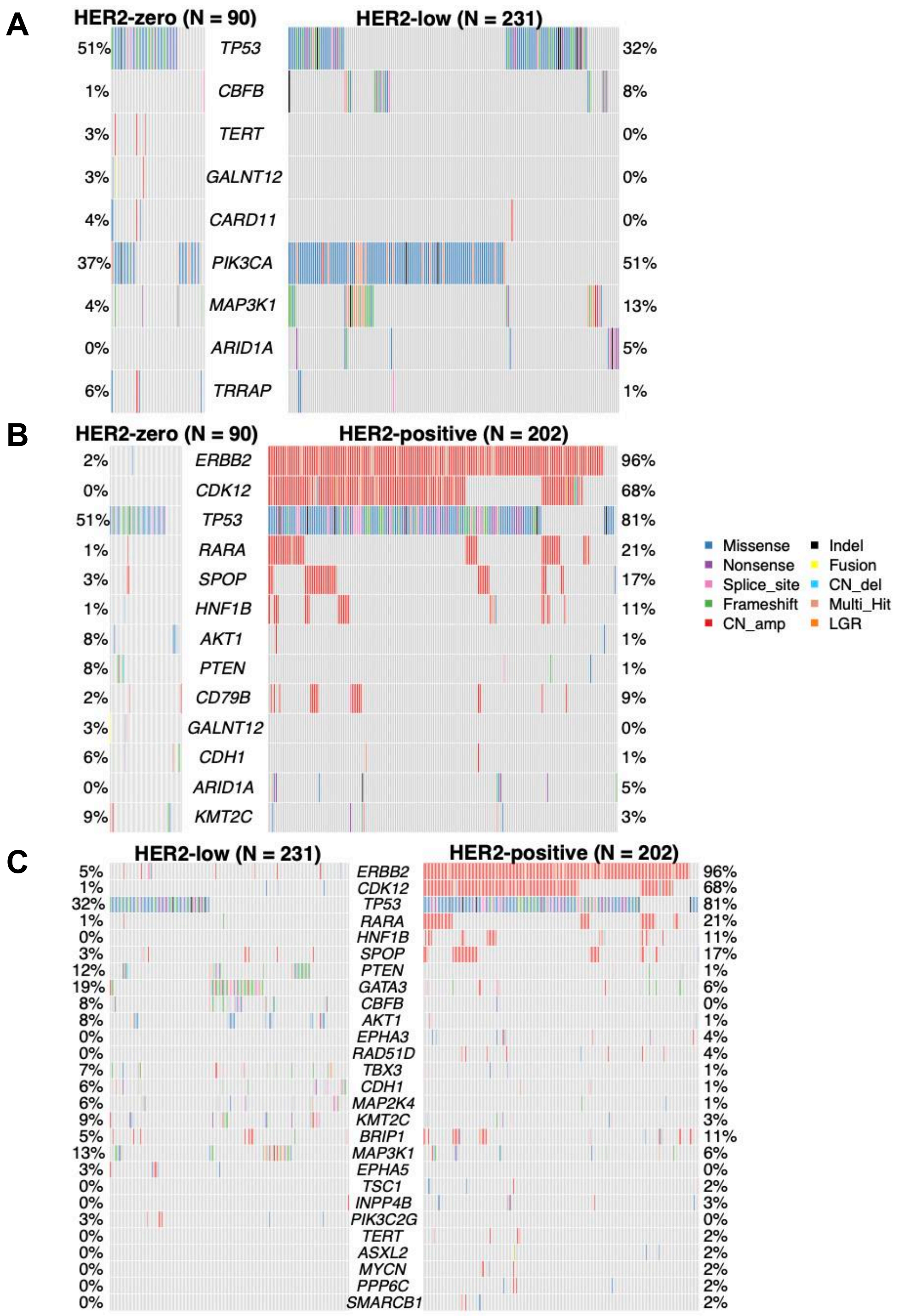

Figure S2. Comparison of mutational profile of the HER2 subgroups. Oncoprints comparing the mutated genes (middle) and mutations types (colors) per patient in HER2-zero (IHC 0) vs HER2-positive (IHC 3+ or IHC 2+/FISH-positive) (**A**), HER2-low (IHC1+ or IHC 2+/FISH-negative) vs HER2-positive (IHC 3+ or IHC 2+/FISH-positive) (**B**), and HER2-zero (IHC 0) vs HER2-low (IHC 1+ or IHC 2+/FISH-negative) (**C**). Mutation detection rates per gene are indicated on the left or right for each subgroup.

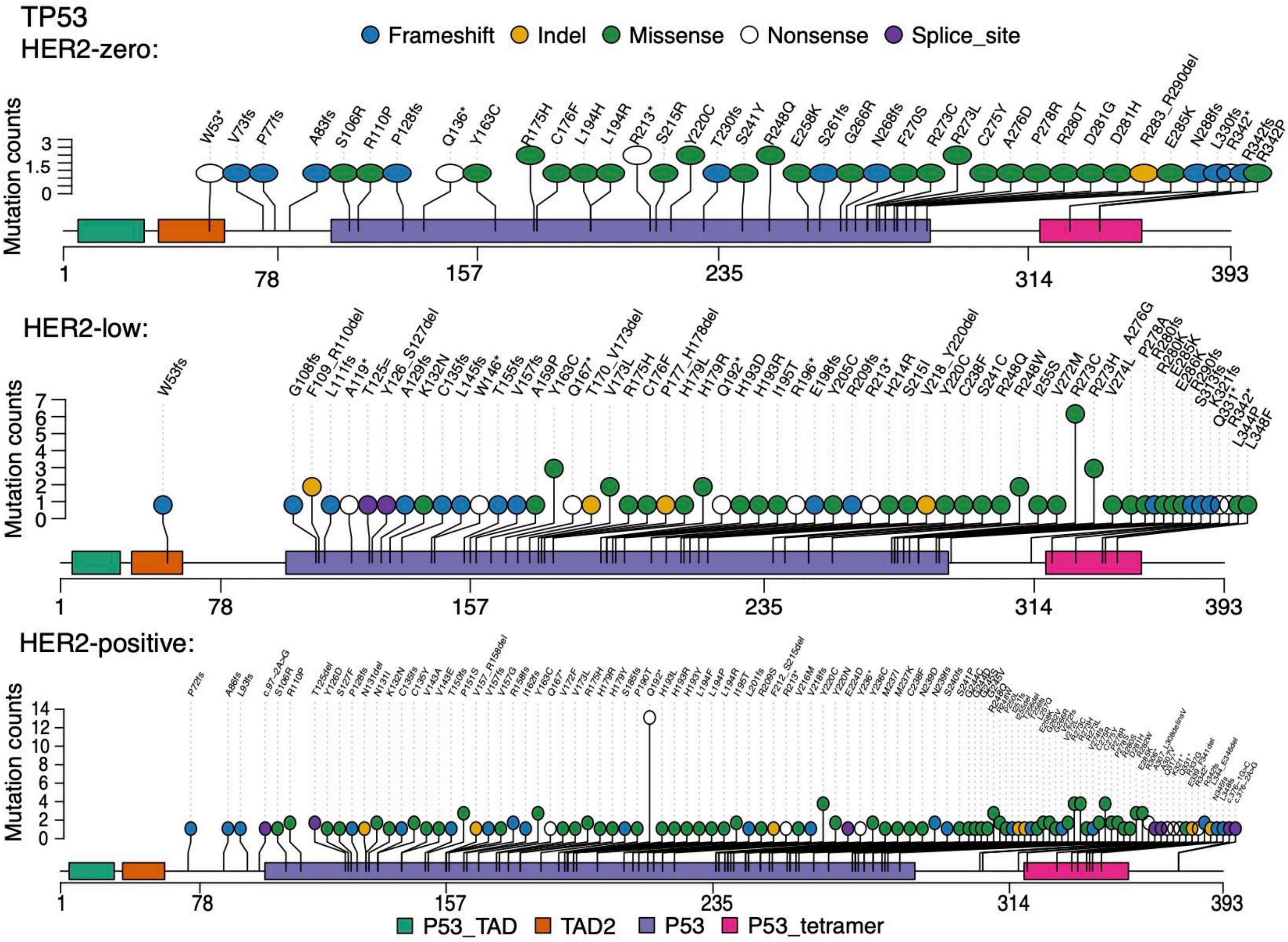

Figure S3. Distribution of *TP53* mutations among the HER2 subgroups.

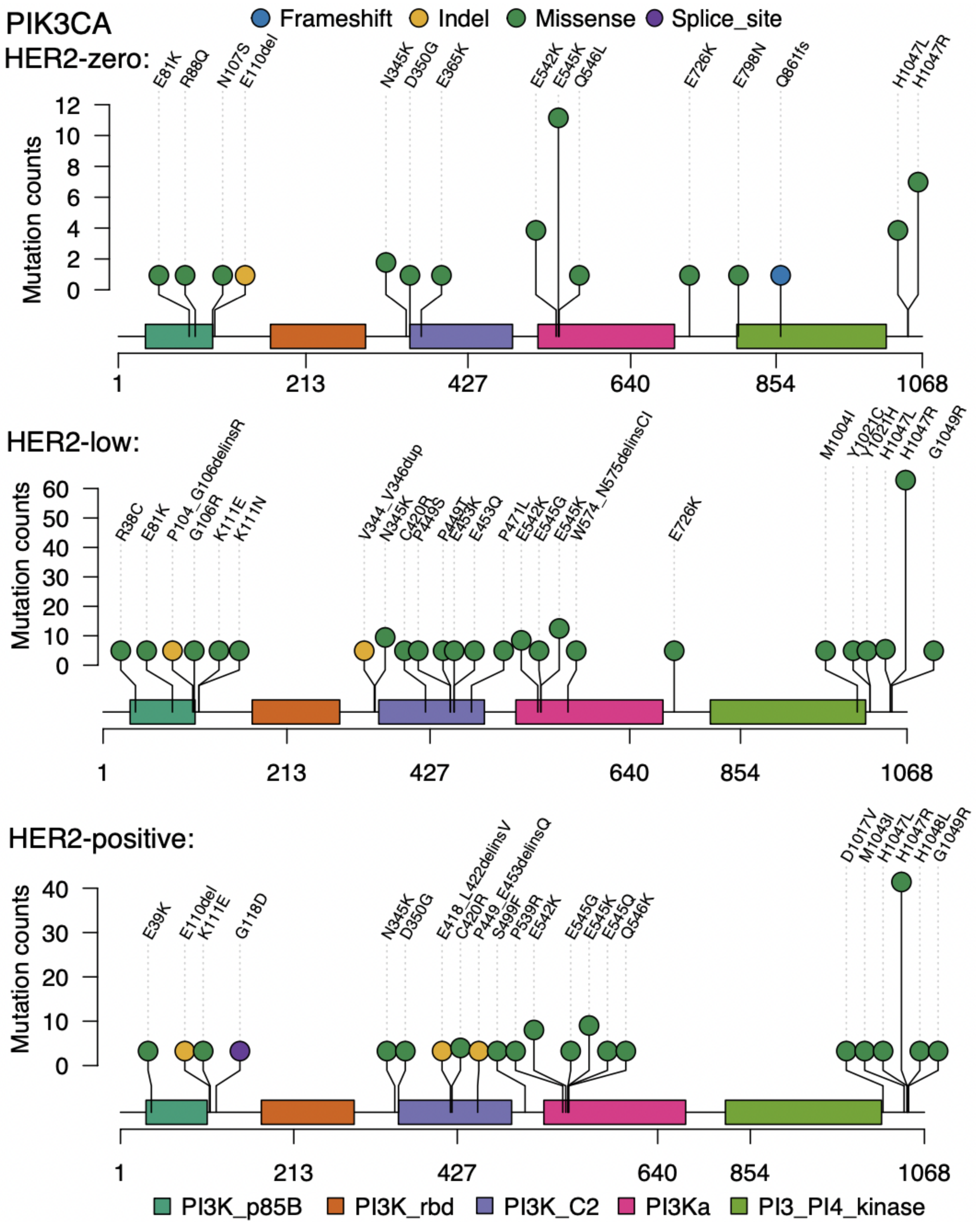

Figure S4. Distribution of *PIK3CA* mutations among the HER2 subgroups.

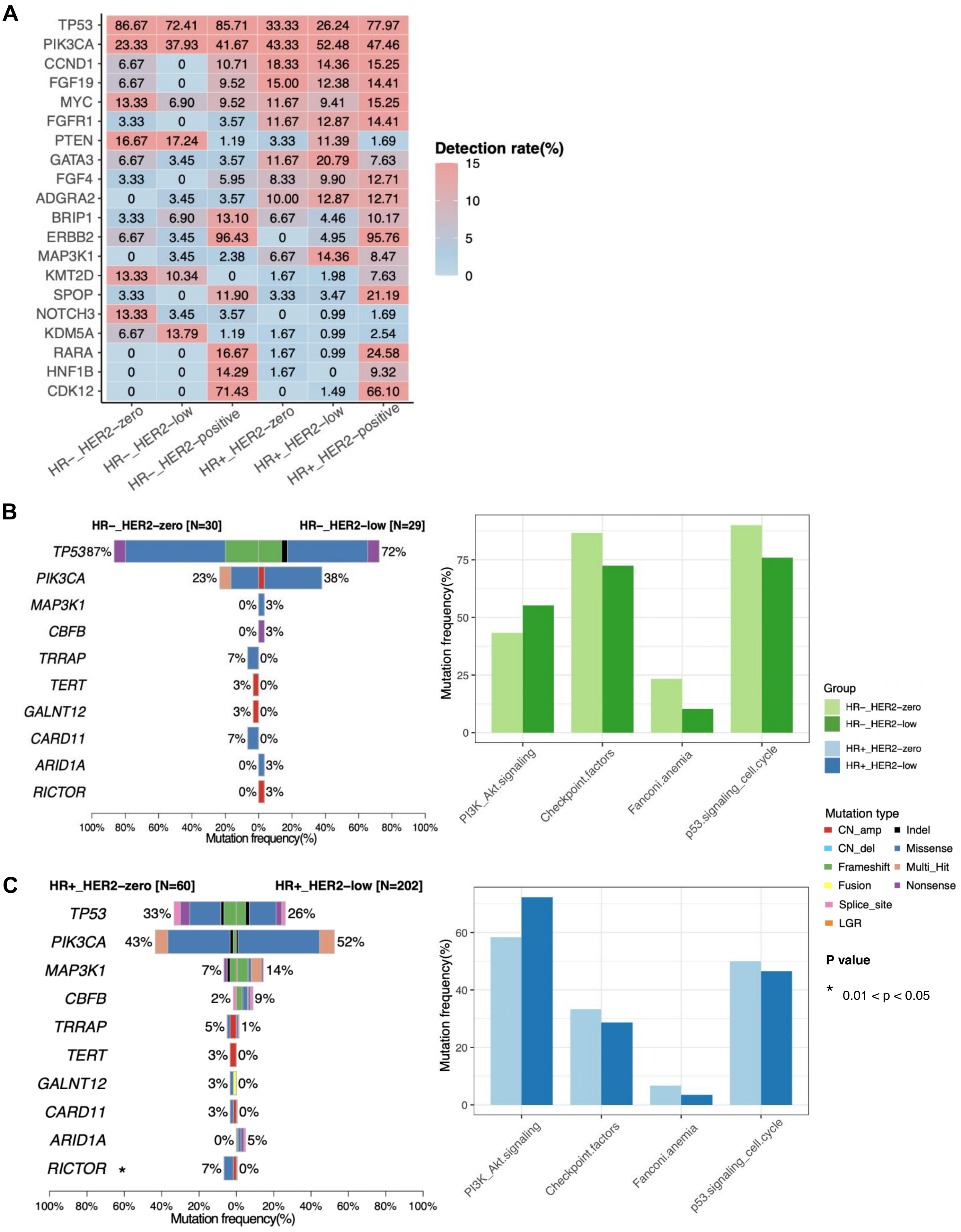

Figure S5. Genes frequently mutated across HER2 subgroups according to hormone receptor (HR) status. **A**. Heat map summarizing the frequently mutated genes across subgroups. **B-C**. Comparison of genes (top 10) and signaling pathways (top 4) differentially mutated between HER2-low and HER2-zero subgroups with HR-negative (HR-) (**B**) and HR-positive (HR+) (**C**) status.

A

B

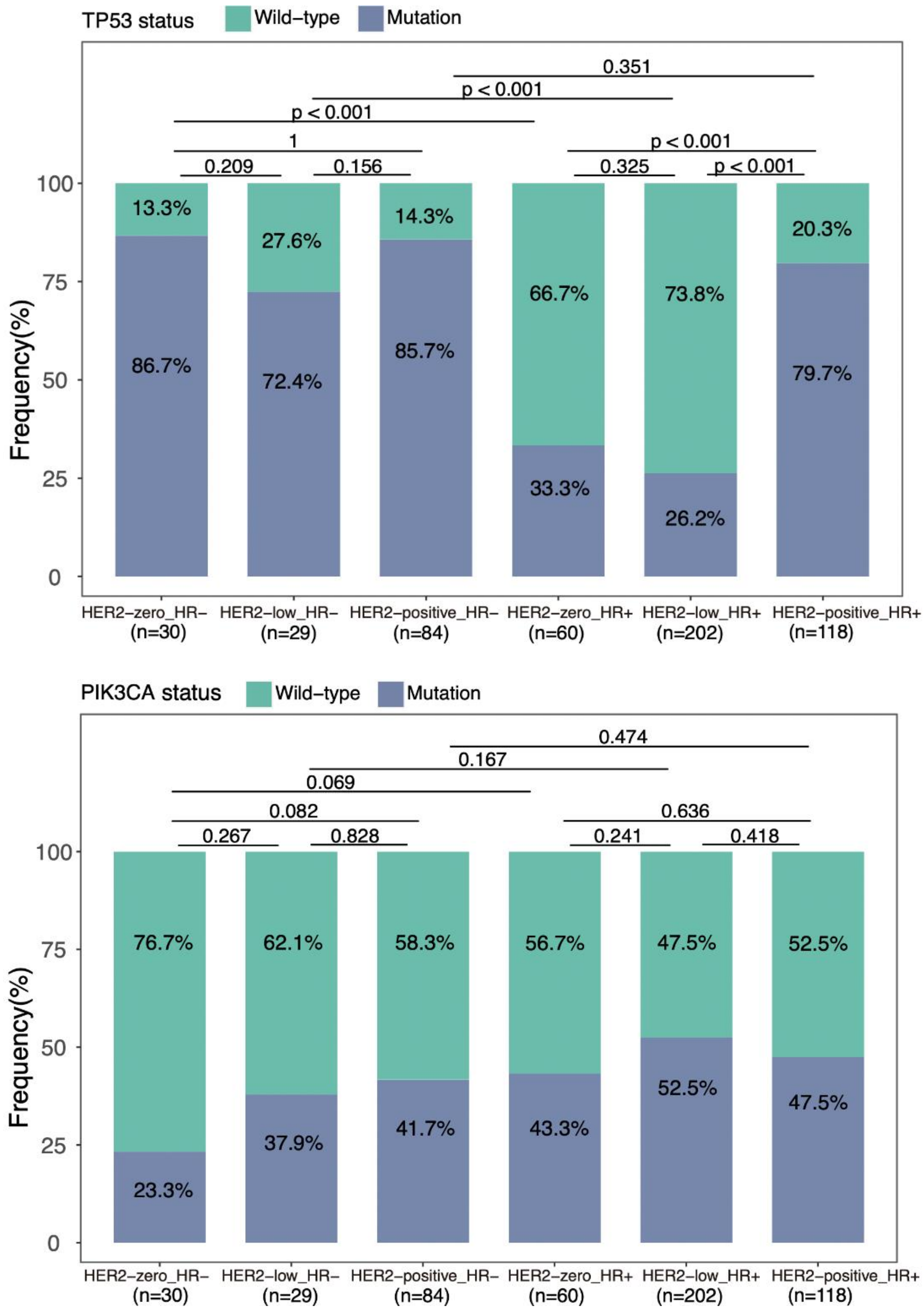

Figure S6. Comparison of *TP53* (**A**) and *PIK3CA* (**B**) mutation rates across HER2 subgroups according to hormone receptor (HR) status.

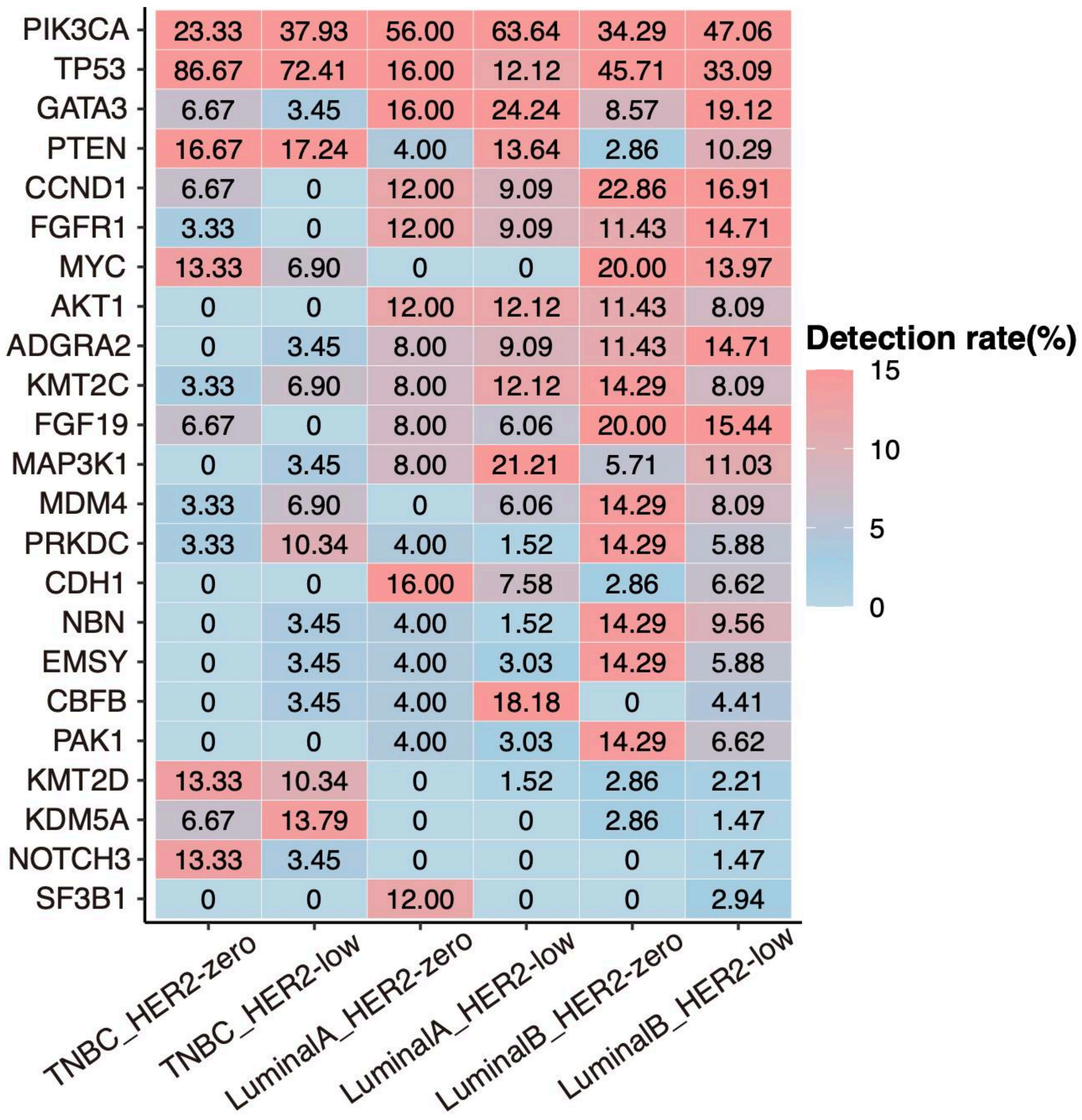

	TNBC_HER2-zero	TNBC_HER2-low	LuminalA_HER2-zero	LuminalA_HER2-low	LuminalB_HER2-zero	LuminalB_HER2-low
PIK3CA	23.33	37.93	56.00	63.64	34.29	47.06
TP53	86.67	72.41	16.00	12.12	45.71	33.09
GATA3	6.67	3.45	16.00	24.24	8.57	19.12
PTEN	16.67	17.24	4.00	13.64	2.86	10.29
CCND1	6.67	0	12.00	9.09	22.86	16.91
FGFR1	3.33	0	12.00	9.09	11.43	14.71
MYC	13.33	6.90	0	0	20.00	13.97
AKT1	0	0	12.00	12.12	11.43	8.09
ADGRA2	0	3.45	8.00	9.09	11.43	14.71
KMT2C	3.33	6.90	8.00	12.12	14.29	8.09
FGF19	6.67	0	8.00	6.06	20.00	15.44
MAP3K1	0	3.45	8.00	21.21	5.71	11.03
MDM4	3.33	6.90	0	6.06	14.29	8.09
PRKDC	3.33	10.34	4.00	1.52	14.29	5.88
CDH1	0	0	16.00	7.58	2.86	6.62
NBN	0	3.45	4.00	1.52	14.29	9.56
EMSY	0	3.45	4.00	3.03	14.29	5.88
CBFB	0	3.45	4.00	18.18	0	4.41
PAK1	0	0	4.00	3.03	14.29	6.62
KMT2D	13.33	10.34	0	1.52	2.86	2.21
KDM5A	6.67	13.79	0	0	2.86	1.47
NOTCH3	13.33	3.45	0	0	0	1.47
SF3B1	0	0	12.00	0	0	2.94

Figure S7. Genes frequently mutated across immunohistochemistry-based molecular subgrouping between HER2-low and HER2-zero subgroups. TNBC, triple negative breast cancer.

A

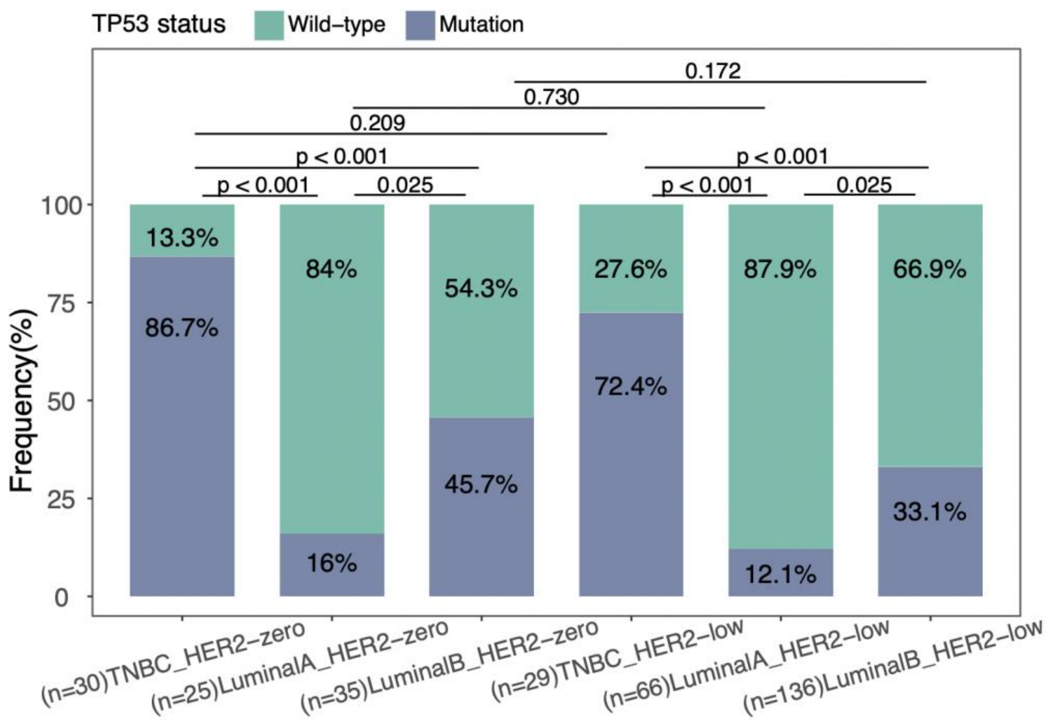

B

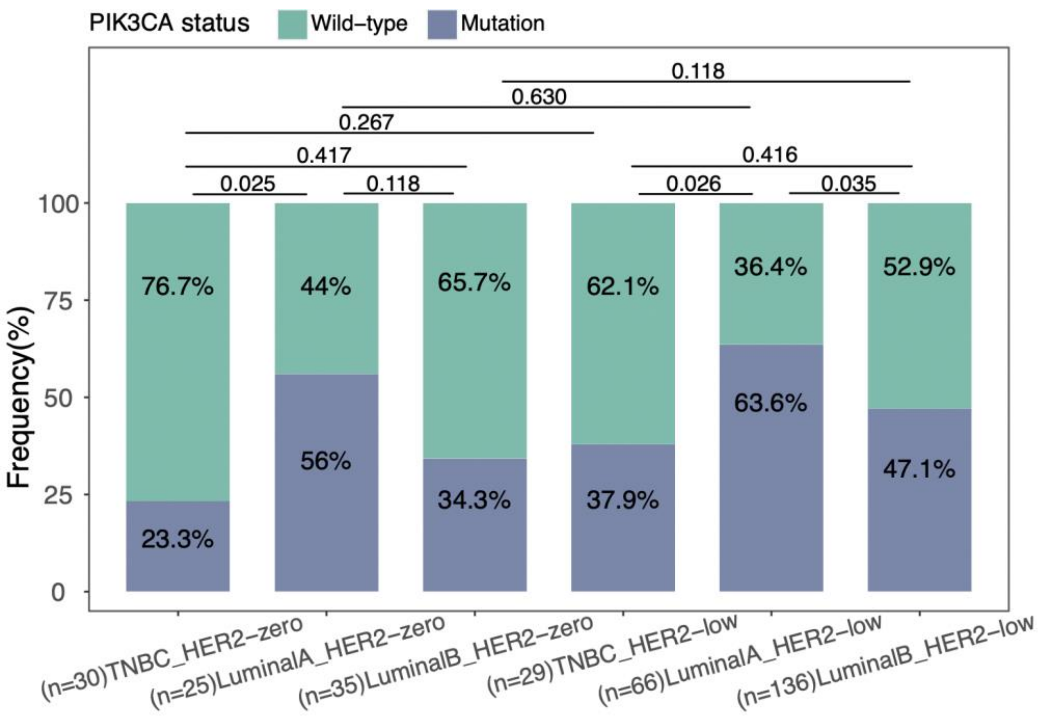

Figure S8. Comparison of *TP53* (**A**) and *PIK3CA* (**B**) mutation rates between HER2-zero and HER2-low subgroups according to immunohistochemistry-based molecular subgrouping. TNBC, triple negative breast cancer.

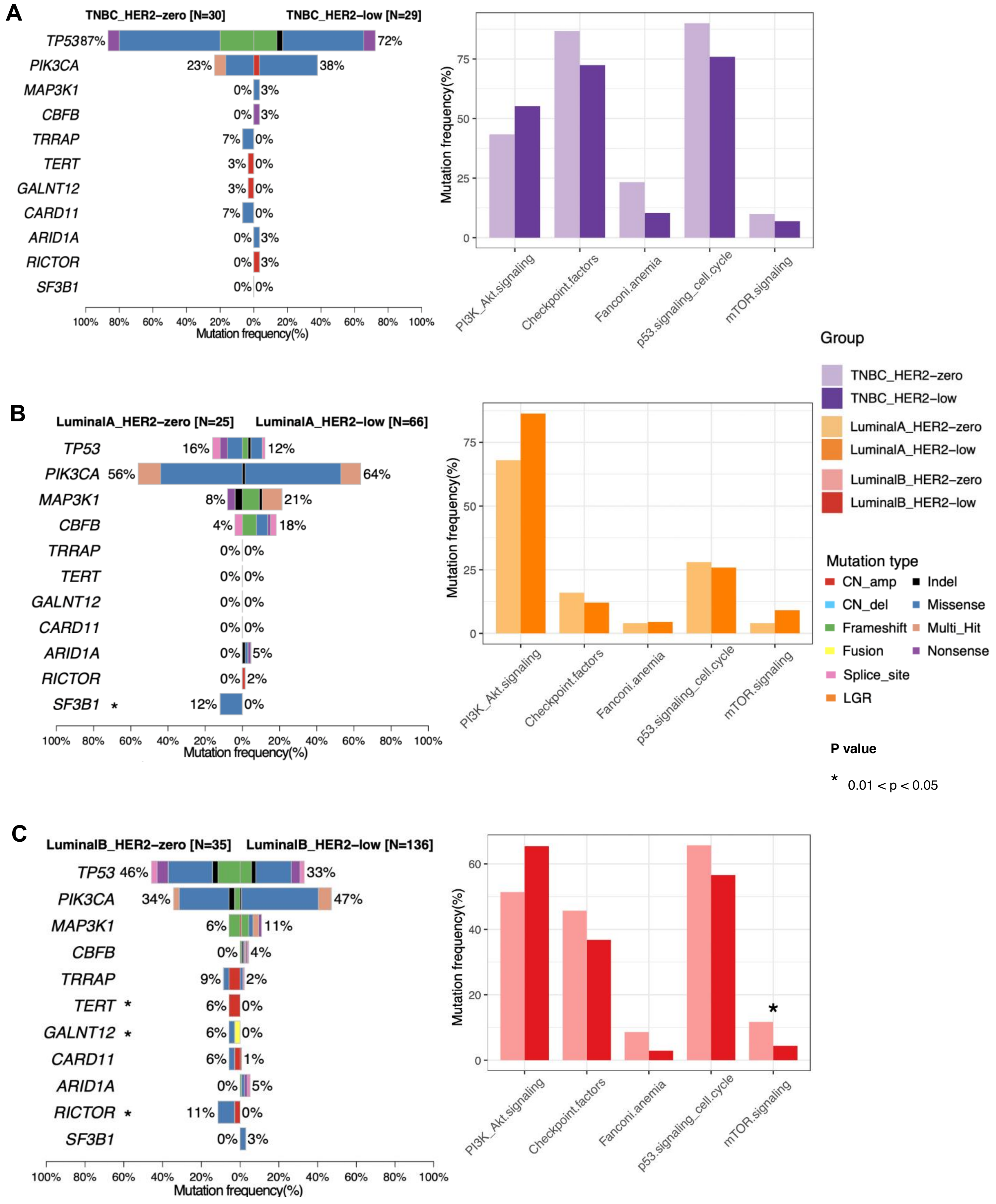

Figure S9. Comparison of genes (top 11) and signaling pathways (top 5) differentially mutated between HER2-low and HER2-zero subgroups according to immunohistochemistry-based molecular subgrouping: **A**. triple negative breast cancer (TNBC), **B**. luminal A, and **C**. luminal B.

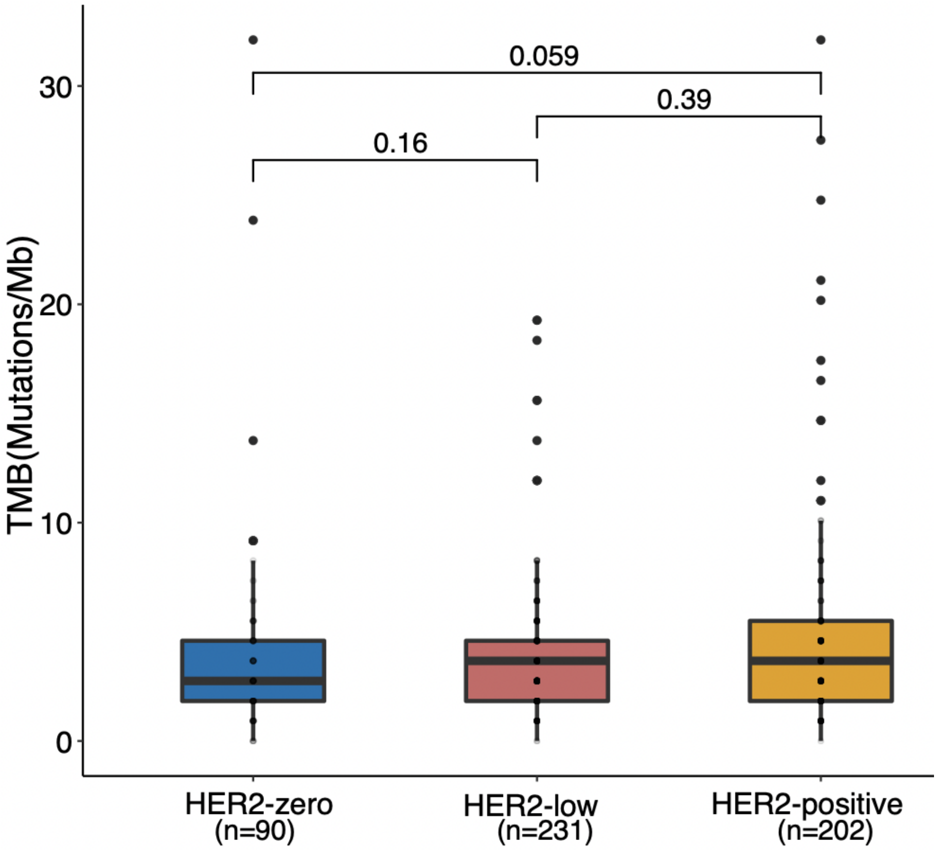

Figure S10. Tumor mutational burden (TMB) were comparable across HER2 subgroups. TMB were not statistically different among patients with HER2-zero (IHC 0), HER2-low (IHC1+ or IHC 2+/FISH-negative), and HER2-positive (IHC 3+ or IHC 2+/FISH-positive) breast tumors.

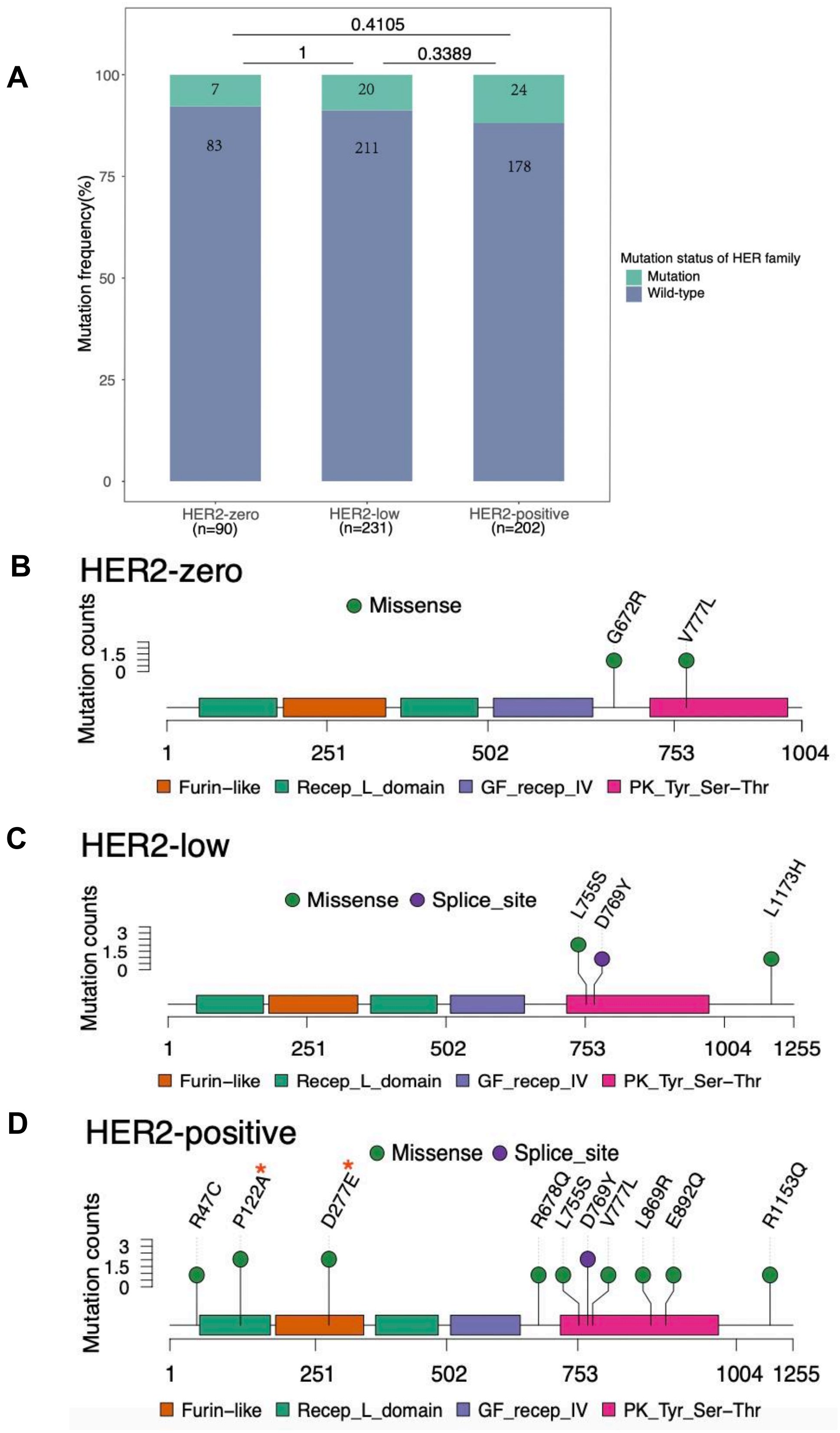

Figure S11. Distribution of ERBB/HER family mutations according to HER2 status. **A.** Mutation rate in ERBB/HER family was comparable among the HER2 subgroups. **B-D**. Lollipop plots summarizing the location of missense or splice-site mutations detected in HER2 gene for each HER2 subgroup.

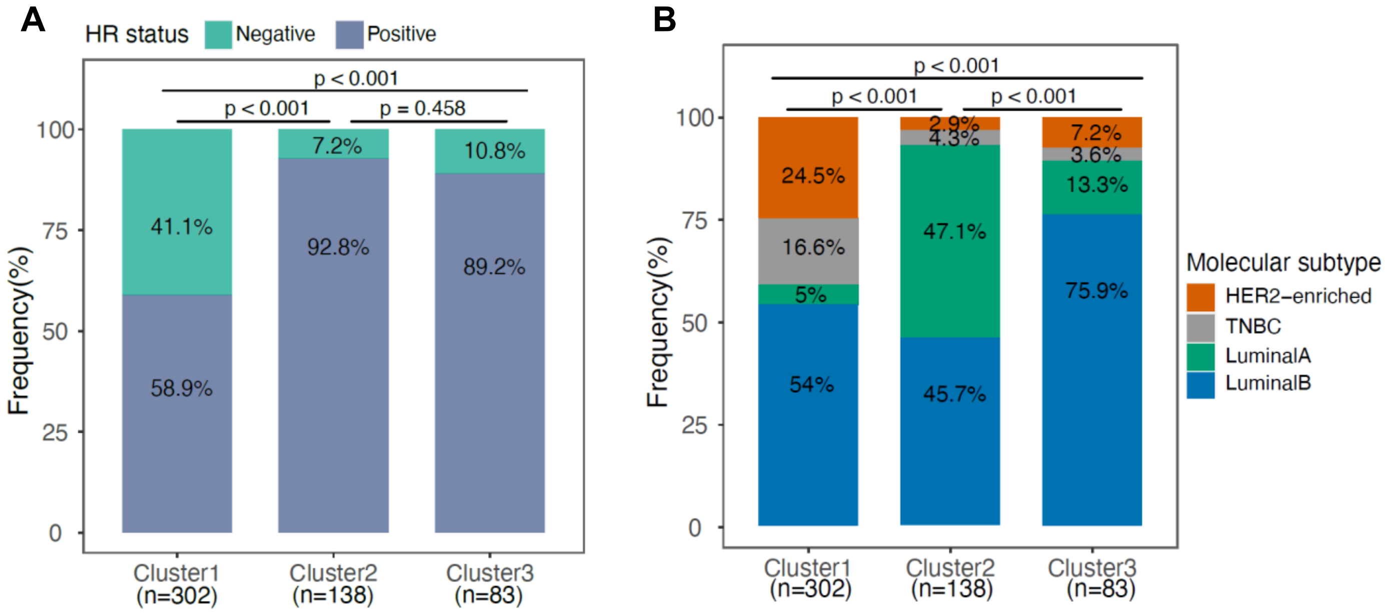

Figure S12. Molecular clustering across hormone receptor (HR) status (**A**) and immunohistochemistry-based molecular subgrouping (**B**). Distribution of HR-positive and HR-negative (**A**) and HER2-enriched, triple negative breast cancer (TNBC), luminal A, and luminal B (**B**) in Cluster 1 (n=302), Cluster 2 (n=138), and Cluster 3 (n=83).

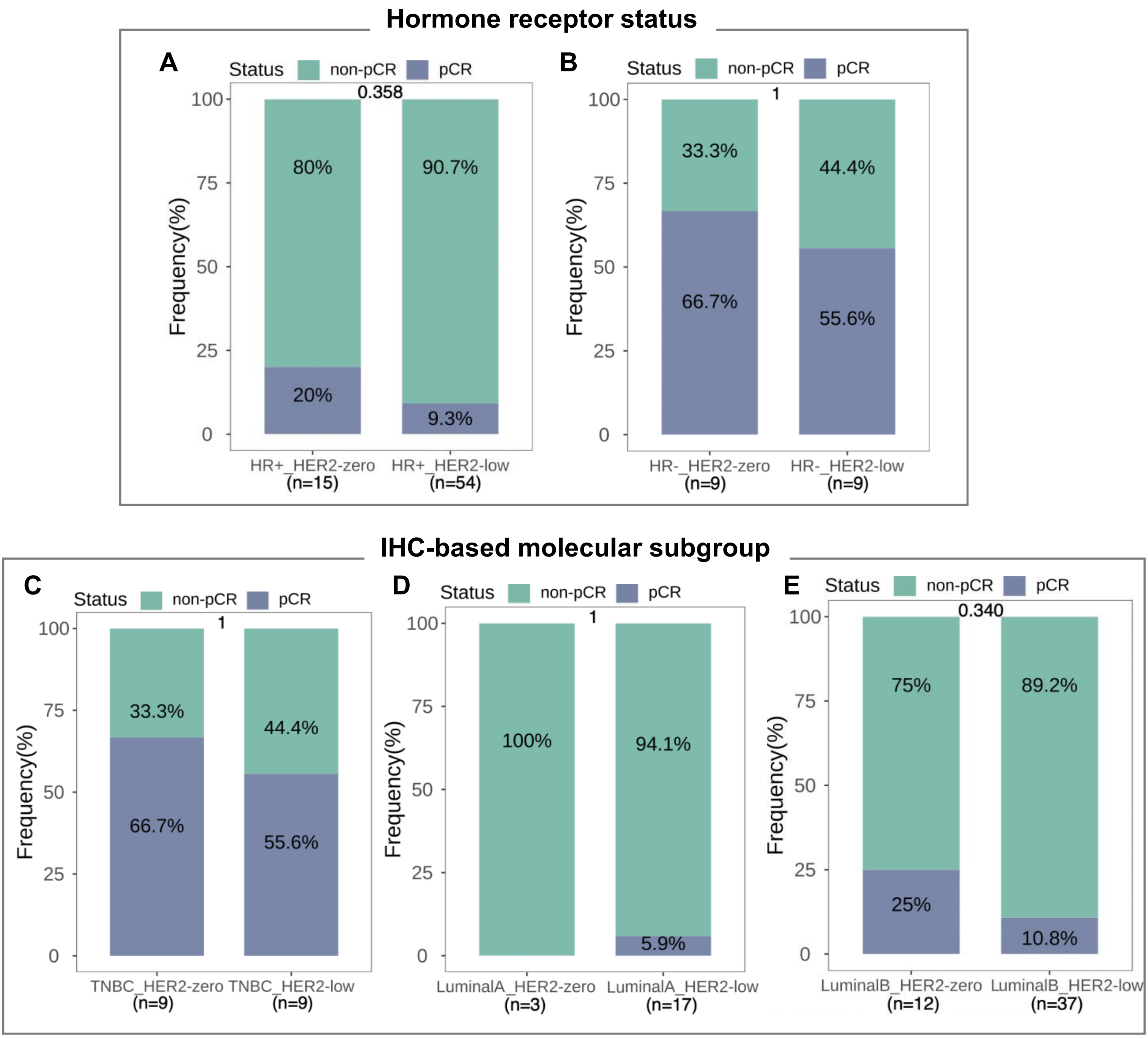

Figure S13. Pathological complete response (pCR) rate is comparable between HER2-low and HER2-zero subgroups across hormone receptor (HR) status (**A-B**) and immunohistochemistry-based molecular subgrouping (**C-E**). Distribution of pCR/non-pCR in HER2-low and HER2-subgroups for HR-positive **(A)**, HR-negative **(B)**, triple negative breast cancer **(C)**, luminal A **(D)**, and luminal B **(E)** breast tumors.

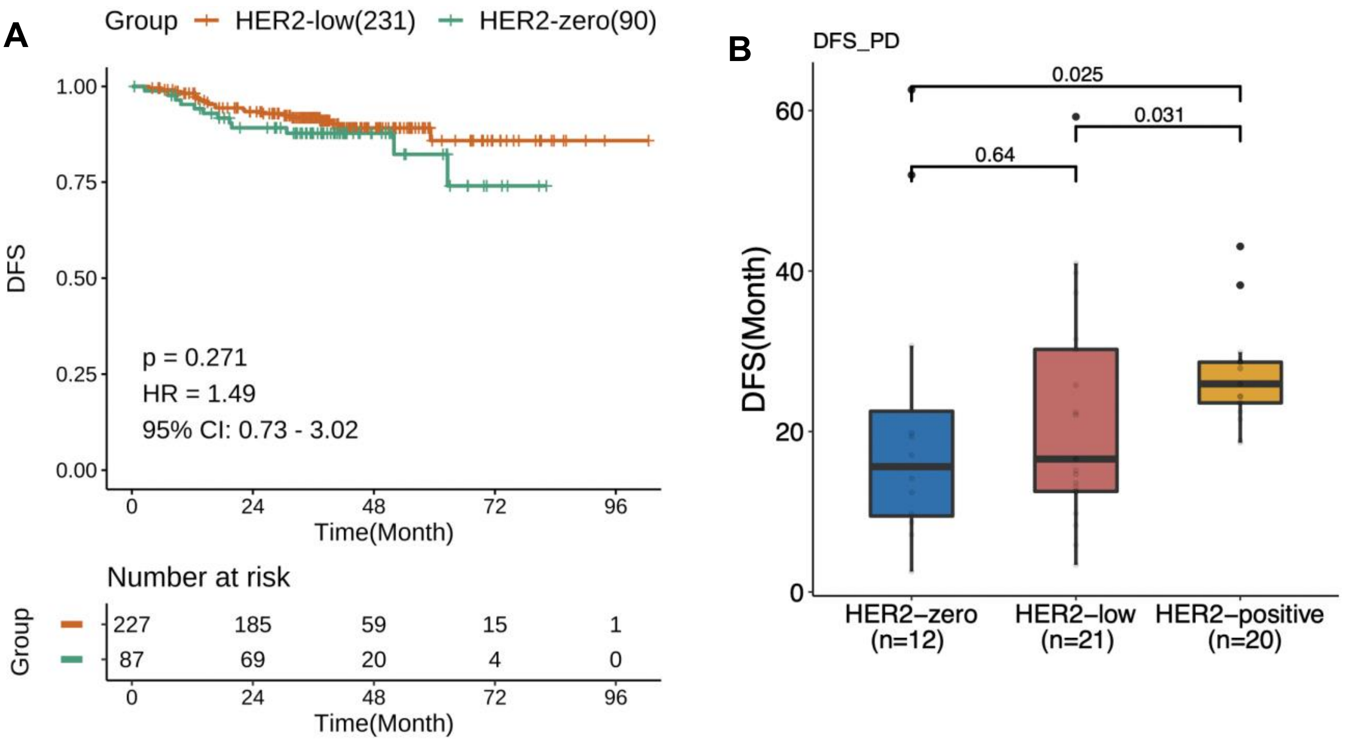

Figure S14. Disease-free survival (DFS) was comparable between women with HER2-low and HER2-zero tumors as shown by Kaplan-Meier survival plot of the whole cohort (**A**) and when considering only the relapsed patients per group (**B**). Tick marks in the survival plot represent censored data (i.e, patients lost to follow-up).

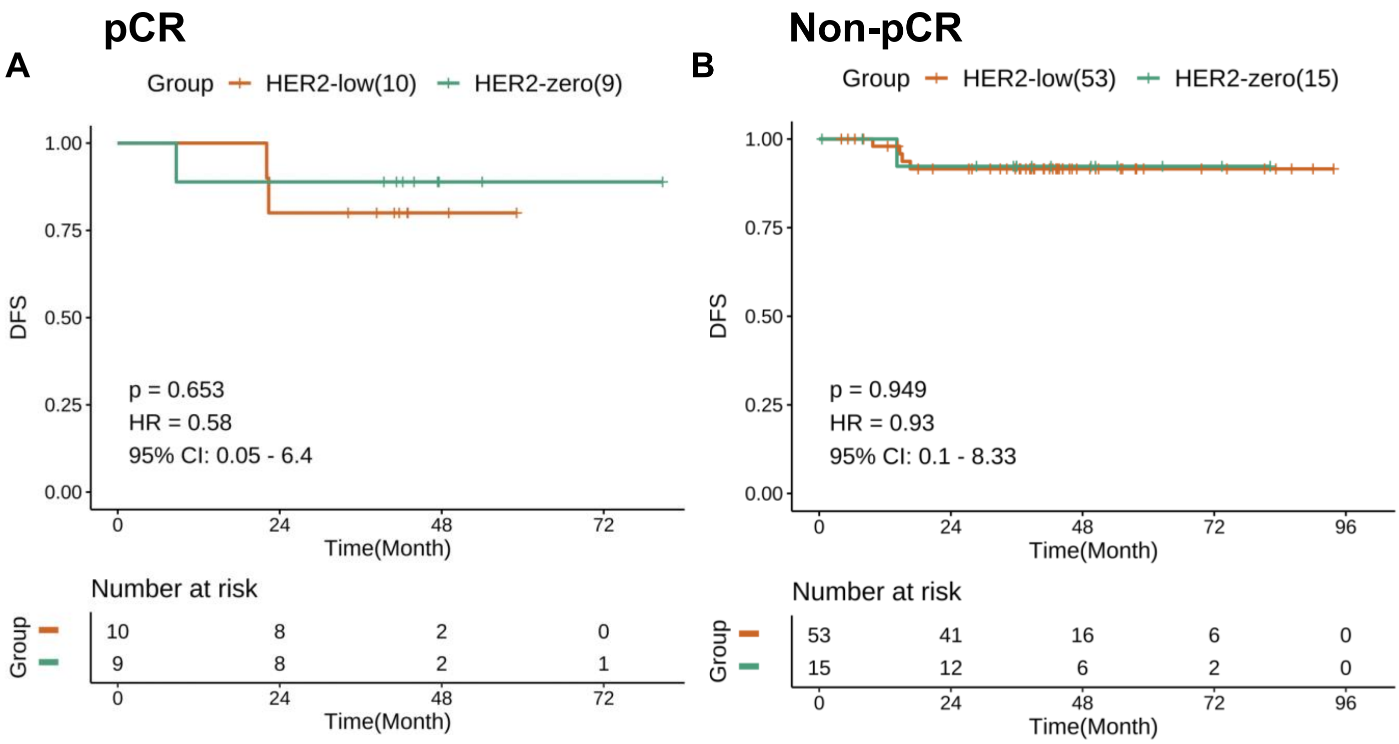

Figure S15. Disease-free survival (DFS) was comparable between HER2-zero and HER2-low subgroups regardless of pathological complete response (pCR) status. Kaplan Meier curves comparing the DFS between women in HER2-zero and HER2-low subgroups who had pCR (**A**) and those who were non-pCR (**B**).

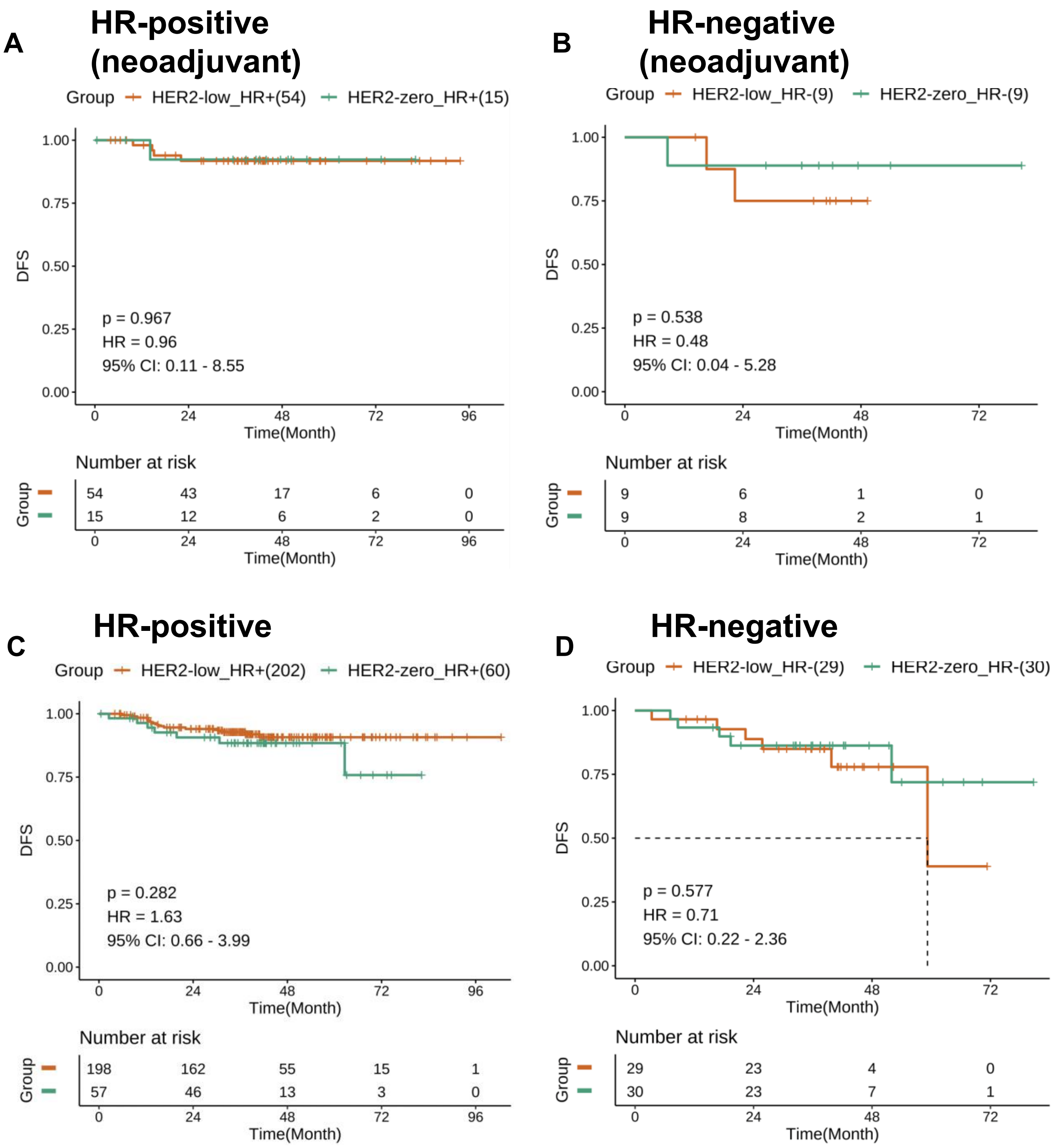

Figure S16. Disease-free survival (DFS) was comparable between HER2-zero and HER2-low subgroups regardless of hormone receptor (HR) status. Kaplan Meier curves comparing the DFS between women in HER2-zero and HER2-low subgroups who had HR-positive tumors (**A, C**) and those who have HR-negative tumors (**B**) among those who received neoadjuvant therapy (**A,B**) and the whole cohort (**C-D**).

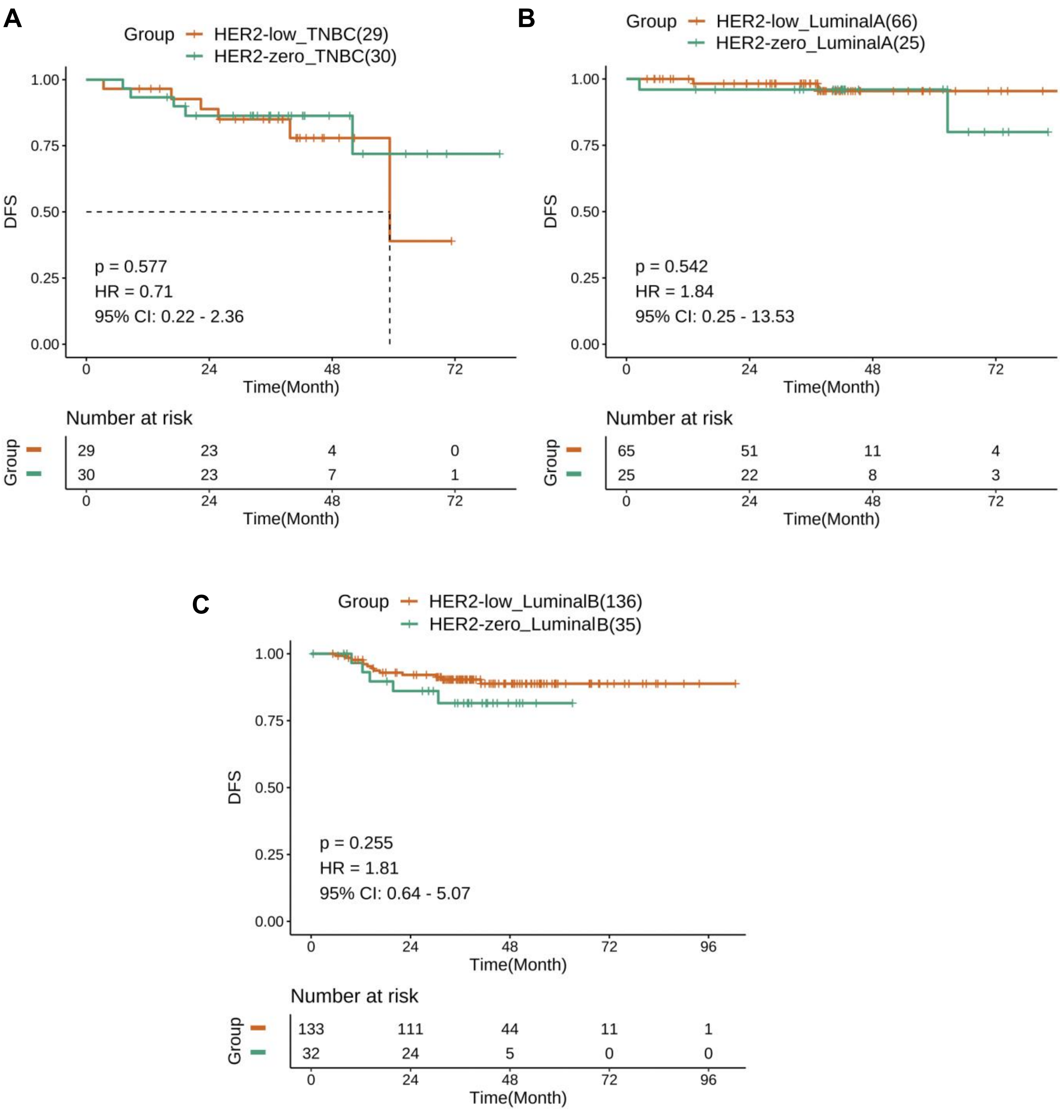

Figure S17. Disease-free survival (DFS) was comparable between HER2-zero and HER2-low subgroups across immunohistochemistry-based molecular subgrouping. Kaplan Meier curves comparing the DFS between women in HER2-zero and HER2-low subgroups who had triple negative breast cancer (**A**), luminal A tumors (**B**), and luminal B tumors (**C**).
